# AZIN1-dependent polyamine synthesis accelerates tumor cell cycle progression and impairs effector T-cell function in osteosarcoma

**DOI:** 10.1038/s41419-025-07640-x

**Published:** 2025-04-17

**Authors:** Jiaming Yu, Chuanxia Zhang, Qinkai Zhang, Bing Lu, Guohao Lu, Chunxiao Zhang, Ru Qiu, Xinyue Wang, Changye Zou, Junjun Chu, Haizhou Li, Wei Zhao

**Affiliations:** 1 Guangdong Cardiovascular Institute, Guangdong Provincial People’s Hospital, Guangdong Academy of Medical Sciences, Guangzhou, 510080 China; 2 Medical Research Institute, Guangdong Provincial People’s Hospital (Guangdong Academy of Medical Sciences), Southern Medical University, Guangzhou, 510080 China; 3 Department of Breast and Thyroid Surgery, Guangzhou Women and Children’s Medical Center, Guangzhou, 510623 China; 4 Center for Stem Cell Biology and Tissue Engineering, Key Laboratory for Stem Cells and Tissue Engineering, Ministry of Education, Sun Yat-sen University, Guangzhou, 510000 China; 5 School of Laboratory Medicine, Guangzhou Health Science College, Guangzhou, 510450 China; 6 Zhongshan School of Medicine, Sun Yat-sen University, Guangzhou, 510000 China; 7 Musculoskeletal Oncology Center, the First Affiliated Hospital of Sun Yat-Sen University, Guangzhou, 510080 China; 8 HRYZ Biotech Co., Guangzhou, 510507 China; 9 Ganzhou Hospital of Guangdong Provincial People’s Hospital, Ganzhou Municipal Hospital, Ganzhou, 341000 China

**Keywords:** Cancer metabolism, Immunosurveillance, Immunotherapy

## Abstract

Osteosarcoma, the most prevalent malignant bone tumor among adolescents, frequently exhibits limited responsiveness to immunotherapy, a challenge attributed to poorly understood underlying mechanisms. Here, we identify enhanced polyamine biosynthesis as a key driver of osteosarcoma progression and immunotherapy resistance. We show that osteosarcoma cell proliferation and tumor growth rely on polyamine availability and that disruption of polyamine synthesis significantly boosts the cytotoxic efficacy of TCR-engineered T cells against osteosarcoma cells. Mechanistically, we reveal that the knockdown of antizyme inhibitor 1 (*AZIN1*) or suppression of polyamine production reduces MYC expression, leading to diminished tumor cell viability via the downregulation of cell cycle-related genes. Furthermore, reduced MYC levels are associated with changes in the expression of immunomodulatory cytokines and human leukocyte antigen molecules, pointing to a potential link with enhanced T-cell-mediated cytotoxicity. Collectively, our findings establish a pivotal role for the AZIN1-polyamine axis in osteosarcoma proliferation and immune evasion, and support the development of novel immunotherapeutic strategies targeting polyamine biosynthesis to combat this aggressive cancer.

## Introduction

Osteosarcoma is the most common malignant bone tumor in adolescents. While conventional treatments, such as radiotherapy and chemotherapy, are effective in approximately 70% of non-metastatic cases, patients who present with metastatic disease at diagnosis face considerably worse five-year survival rates, underscoring the need for improved therapeutic strategies [1, 2]. Although immunotherapy has emerged as a promising approach for various cancers, its success in osteosarcoma has been limited [3, 4]. This limitation highlights the importance of gaining a deeper understanding of the tumor’s immune microenvironment.

Recent studies have emphasized the profound impact of metabolic dysregulation on immunotherapy effectiveness [5]. For instance, mutations in isocitrate dehydrogenase (IDH), which lead to elevated levels of the oncometabolite 2-hydroxyglutarate (2HG), can markedly influence immune responses [6]. However, the full spectrum of metabolic alterations in osteosarcoma remains insufficiently characterized. Of particular interest, polyamine metabolism (encompassing putrescine, spermidine, and spermine) plays an essential role in proliferation, differentiation, apoptosis, and immune regulation [7, 8]. Despite the recognized significance of these metabolites, their specific impact on immunotherapeutic outcomes in osteosarcoma has yet to be thoroughly investigated.

A central enzyme in polyamine biosynthesis is ornithine decarboxylase (ODC1), which converts ornithine into putrescine and serves as the rate-limiting step in this pathway [9]. Notably, ODC1 activity is regulated by antizyme inhibitor 1 (AZIN1), which stabilizes ODC1 by inhibiting antizyme-mediated degradation [10]. This regulatory axis presents a potential target for modulating polyamine levels in tumor cells. However, a comprehensive picture of the metabolic profiles and regulatory mechanisms controlling polyamine dynamics in osteosarcoma remains incomplete.

In this study, we investigated the metabolic landscape of osteosarcoma and uncovered a dysregulated polyamine biosynthesis pathway driving both tumor cell proliferation and immune evasion. Mechanistically, we identified AZIN1 as a key regulator of this process. Reducing AZIN1 levels diminished MYC expression and suppressed cell cycle progression, ultimately inhibiting tumor growth. Furthermore, reduced MYC levels are associated with increased human leukocyte antigen (HLA) expression, suggesting a potential link to enhanced T-cell-mediated cytotoxicity. Collectively, these findings underscore the pivotal role of the AZIN1-polyamine axis in osteosarcoma and support developing immunotherapeutic strategies that target polyamine metabolism to overcome resistance and enhance treatment efficacy.

## Materials and methods

### Collection of osteosarcoma specimens and adjacent normal tissue

Osteosarcoma specimens and their corresponding normal adjacent tissues were collected during surgical procedures at the Department of Bone Tumor, the First Affiliated Hospital of Sun Yat-sen University, following protocols approved by the institutional review board. Written informed consent was obtained from each participant after providing a detailed explanation of the procedure and its associated risks, in compliance with the Declaration of Helsinki. All collected specimens were subjected to histopathological examination by a qualified pathologist to confirm the tumor type and grade.

### Non-targeted metabolomics

Osteosarcoma adjacent normal tissues were retrieved from liquid nitrogen storage and thawed on ice before being weighed. Samples of 50 mg were transferred into 2 mL centrifuge tubes, to which 800 µL of 80% methanol was added. The samples were homogenized for 90s, vortexed for one minute, and then ultrasonicated for 30 min at 4 ℃. After ultrasonication, samples were incubated at -40 ℃ for one hour, vortexed again for 30 s, and allowed to settle at 4 ℃ for 30 min without disturbance. Following this, the samples were mixed by inversion and centrifuged at 12,000×*g* at 4 ℃ for 15 min. Subsequently, 200 µL of the supernatant was transferred to an autosampler vial for LC–MS non-targeted metabolomics analysis conducted by Shanghai Sensichip Infotech Co., Shanghai, China.

Raw data were processed using Compound Discoverer 3.1 (CD) software to perform multivariate statistical analyses. These analyses included qualitative and quantitative assessments, quality control measures, orthogonal projections to latent structures-discriminant analysis (OPLS-DA), and differential metabolite analysis. Results from the differential metabolite analysis were subjected to pathway enrichment analysis using MetaboAnalyst 6.0 (http://www.metaboanalyst.ca). *P*-values less than 0.05 were considered statistically significant. Details of metabolite abundance are provided in Table S3.

### Animals

Female Balb/c nude mice and NSFG mice (NOD-Prkdc^scid^-B6-IL2rg^nullNh^), weighing 18 ± 2 g and aged 4–6 weeks, were procured from the Model Animal Research Center of Nanjing University and RUIYE Laboratory, Guangzhou, China, respectively. All experimental procedures involving these animals were conducted in strict accordance with the guidelines approved by the Ethical Committee of the First Affiliated Hospital of Sun Yat-sen University.

### Orthotopic xenograft model

A single-cell suspension of osteosarcoma cells (20 µL) containing 1–5 × 10^5^ cells was orthotopically injected into the right tibial medulla of 5-week-old nude mice or NSFG mice, which were anesthetized using chloral hydrate. A customized arginine-deprived mouse diet was obtained from MolDiets, Beijing, China. 0.5% DFMO drinking water was prepared by dissolving 1 g of DFMO (Invivochem) with 200 mL sterile water and sterilized using a 0.22 μm PES (polyethersulfone) filter. Three to six weeks post-injection, tumor growth was monitored using an in vivo imaging system (IVIS, Xenogen) under isoflurane anesthesia. After orthotopic tumors became visible, their dimensions were measured with calipers to record both length and width. Tumor volumes were calculated using the formula: volume (mm^3^) = (length × width^2^)/2. When the tumor volumes reached 20–50 mm^3^, mice were randomly allocated to either TCR-T cell treatment groups or control groups receiving non-transduced T cells, ensuring an equal initial tumor burden. Mice with tumors smaller than 20 mm^3^ were excluded from the study. The maximal tumor (2 cm diameter) size/burden was not exceeded in all experiments.

### Cell culture methods

Human osteosarcoma cell lines 143B, U2-OS, SJSA-1, MNNG, and human embryonic kidney 293T cells were maintained in DMEM medium (Corning, USA) supplemented with 10% fetal bovine serum (FBS) (BI, USA). Customized arginine-deprived DMEM medium was obtained from BOSTER Biological Technology, Ltd. (Wuhan, China). Cultures were incubated at 37 ℃ in a humidified atmosphere containing 5% CO_2_. Human peripheral blood mononuclear cells (PBMCs) obtained from LDEBIO, Guangzhou, along with T cells, were cultured in a T cell medium (TCM). This medium consisted of RPMI-1640 (Gibco), enriched with 10% FBS (BI, USA), 25 mM HEPES (Corning), 2 mM GlutaMax (Gibco), 50 µM β-mercaptoethanol (Sigma), and 50 IU/mL recombinant human Interleukin-2 (rhIL-2) (Peprotech).

### Cell viability assay

Cell proliferation was quantified using the CellTiter-Glo® Luminescent Cell Viability Assay kit (Promega), following the manufacturer’s protocol. Briefly, 2000 cells suspended in 100 µL of complete culture medium were seeded into each well of a 96-well culture plate. At 24, 48, 72, and 96 h post-inoculation, an equal volume of CellTiter-Glo® Reagent was added to each well to lyse the cells. The contents were then transferred to an opaque 96-well plate, and luminescence was measured using a Biotek Synergy 2 plate reader.

### Construction of plasmids and viral packaging

Short hairpin RNAs (shRNAs) targeting specific genes were cloned into the pLVX-puro lentiviral vector. The MAGE-A4/HLA-A*02:01 specific TCR sequence was obtained from a patent (WO 2017/174824 A1) held by Adaptimmune Ltd. For lentiviral particle production, the plasmids expressing target sequences were co-transfected with the packaging plasmids pVSV-G and psPAX2 into human embryonic kidney 293T cells using StarFect II Transfection Reagent (GenStar, China). The medium containing lentiviral particles was collected at 36 and 72 h post-transfection. The sequences of the shRNA targets are provided in Table S1. Additionally, lentiviral particles T cells transduction were concentrated 50-fold by centrifugation at 3000*g* for 1 h at 4 ℃ with 8.5% PEG6000 and 0.3 M NaCl. Particle pellets were resuspended in TCM (without rhIL-2) and stored at −80 ℃ until required.

### Western blotting (WB)

Cells were lysed in RIPA buffer (50 mM Tris-HCl, pH 7.4, 150 mM NaCl, 1% NP-40, 0.5% sodium deoxycholate, 0.1% SDS) supplemented with protease and phosphatase inhibitors (Roche). The lysates were incubated on ice for 30 min and then clarified by centrifugation at 12,000*g* for 15 min at 4 ℃. The protein concentration in the supernatant was determined using the BCA Protein Assay Kit (Thermo Fisher Scientific). Equal amounts of protein (20 μg) were resolved by SDS-PAGE on a 10% polyacrylamide gel and transferred to a polyvinylidene difluoride (PVDF) membrane (Millipore). Membranes were blocked in 5% non-fat dry milk in Tris-buffered saline with 0.1% Tween-20 (TBST) for 1 h at room temperature. The membranes were then incubated overnight at 4 ℃ with primary antibodies diluted in 5% bovine serum albumin (BSA) in TBST. After washing three times with TBST, the membranes were incubated with horseradish peroxidase (HRP)-conjugated secondary antibodies diluted in 5% non-fat dry milk in TBST for 1 h at room temperature. Following three additional washes with TBST, the protein bands were visualized using an enhanced chemiluminescence (ECL) detection system (Thermo Fisher Scientific) and imaged using a ChemiDoc MP Imaging System (Bio-Rad). The primary antibodies used in this study included CDK2 (78B2) Rabbit mAb (#2546, CST), Cyclin D1 (92G2) Rabbit mAb (#2978, CST), p21 Waf1/Cip1 (12D1) Rabbit mAb (#2947, CST), CDK4 (D9G3E) Rabbit mAb (#12790, CST), p18 INK4C (DCS118) Mouse mAb (#2896, CST), Phospho-RB-S807/811 Rabbit pAb (#AP0484, Abclonal), Mouse monoclonal anti-β-actin (#60008, Proteintech), and Anti-PD-L1 antibody [EPR19759] (#ab213524, abcam).

### Quantitative real-time PCR (qPCR)

Total RNA was extracted from cells using TRIzol reagent according to the manufacturer’s instructions. cDNA was generated from mRNA using HiScript II Q RT SuperMix (#R223, Vazyme) following the removal of gDNA according to the manufacturer’s instructions. Quantitative real-time PCR was carried out on the Applied Biosystems QuantStudio 6 Flex Real-Time PCR system (Thermo Fisher Scientific) with ChamQ Universal SYBR qPCR Master Mix (#Q711, Vazyme). The primers used are shown in Table S2.

### Human T cell transduction

Primary human T cells were activated ex vivo by seeding peripheral blood mononuclear cells (PBMCs) in an anti-CD3 (OKT3) antibody-coated plate along with anti-CD28 antibody (1 µg/mL) in T cell medium (TCM) for two days. Following activation, the cells were pelleted and resuspended at a concentration of 5 × 10^5^ cells/mL in fresh TCM supplemented with lentiviral supernatant and 8 µg/mL polybrene. Transduction efficiency was assessed three days post-transduction.

### Flow cytometry

For the detection of MAGE-A4/HLA-A*02:01 (A2M4) specific TCR transduction efficiency, transduced T cells were washed once with 1% BSA-PBS wash buffer and stained with PerCP-Cyanine5.5 anti-human TCR Vα24 antibody (Biolegend) at the recommended dilution, following the manufacturer’s instructions. Non-transduced, activated T cells stained with the same antibody were used as negative controls. For cell cycle analysis, 30 μM BrdU was added to the culture medium and incubated at 37 ℃ for 3 h. After incubation, cells were washed twice with PBS, dissociated into single cells, and fixed in 70% ethanol. BrdU staining was performed using the FITC-BrdU Cell Proliferation Detection Kit (#B502, Solarbio; #KGA9201, KeyGEN BioTECH, China), following the manufacturer’s instructions. For HLA-A/B/C and CD3 staining, tumors were harvested 7 days after 0.5% DFMO treatment and 3 days after A2M4-TCR T cells injection, respectively. Tumors were cut into pieces, digested with 1 mg/mL collagenase Type IV (#17104019, Gibco) and 20 µg/mL DNaseI (#1121MG010, Biofroxx), ground and filtered using 70 µm cell strainers (#CSS013070, JETBIOFIL). Single cells were stained with Tonbo™ Ghost Dye™ Violet 510 (#13-0870-T100, eBioscience), fixed in 4% paraformaldehyde solution, blocked with mouse IgG, and stained with antibodies including HLA class I A/B/C antibody (#66013-1-Ig, proteintech), Goat anti-mouse IgG Alexa Fluor™ 488 (#A28175, Invitrogen) and anti-Human CD3-APC (#F1100303, MULTI SCIENCES). Flow cytometry analysis was conducted using a CytoFLEX instrument (Beckman Coulter).

### In vitro cytotoxicity assay

U2-OS or 143B cells expressing HLA-A*02:01 and MAGE-A4 were transduced with either control shRNA or shRNA targeting AZIN1, or treated with PBS or 5 mM DFMO for 48 h prior to co-incubation with TCR-T cells. Both tumor cells and TCR-T cells were washed once with PBS and co-cultured at specified TCR-T cells-to-tumor cells ratios (E:T ratio) in T cell assay medium (TAM). TAM consists of TCM supplemented with 5% FBS but lacks IL-2. The co-culture was maintained for approximately 16 h. For PD-L1 blocking experiments, tumor cells were pre-treated with 10 µg/mL anti-PD-L1 antibody (Atezolizumab, #B2016, BIOINTRON) or an isotype control antibody at 37 ℃ for one hour prior to co-culturing with TCR-T cells. Specific cytotoxicity was assessed by measuring lactate dehydrogenase (LDH) release using the LDH Cytotoxicity Assay Kit (Beyotime), following the manufacturer’s instructions.

### ELISA

The levels of arginine, ornithine, putrescine, spermidine, and IFN-γ were quantified using ELISA kits (MEIMIAN and Cloud-Clone Corp., China). For metabolite detection, tumor cells were harvested, washed three times with PBS, resuspended in PBS, and lysed by sonication. Cellular debris was removed by centrifugation, and the supernatant was stored at −20 ℃ until further analysis. For IFN-γ detection, the co-culture medium was centrifuged at 500×*g* for 5 min, and the supernatant was collected and stored at −20 ℃ until analysis. On the day of the experiment, all reagents and samples were equilibrated to room temperature. A total of 50 μL of each sample was added to ELISA wells in duplicate, and subsequent steps were performed according to the manufacturer’s instructions. Absorbance was measured at 450 nm using a Synergy2 plate reader (BioTek).

### RNA sequencing

Total RNA was extracted using TRIzol reagent (Life Technologies, USA) according to the manufacturer’s instructions and quantified using a Nanodrop 2000 spectrophotometer. RNA integrity was assessed using the Agilent Bioanalyzer 2100. Paired-end sequencing was performed by Novogene (Novogene, China) using the Illumina NovaSeq X Plus platform. Raw sequencing reads were cleaned using Trim Galore to remove low-quality reads and adapter sequences. Cleaned reads were then aligned to the human reference genome (build hg38) using HISAT2 software. Differential expression analysis was performed using DESeq2, with a significance threshold of *p* < 0.05. Gene set enrichment analysis (GSEA) was conducted using the clusterProfiler, ReactomePA, and enrichplot packages to identify enriched pathways.

### Quantification and statistical analysis

All in vitro experiments were conducted in at least triplicate. Statistical analyses were performed using GraphPad Prism version 8.0 (GraphPad Software, USA). Data are presented as mean ± SD. For comparisons between two groups, unpaired or paired two-tailed t-tests were used to assess statistical significance. For comparisons involving three or more groups, one-way or two-way ANOVA was applied. *P*-values less than 0.05 were considered statistically significant and denoted as ‘*’; *p*-values less than 0.01 and 0.001 were indicated as ‘**’ and ‘***’, respectively. Non-significant results were labeled as “n.s.”

## Results

### Association of elevated polyamine synthesis with osteosarcoma progression

**Fig. 1:**
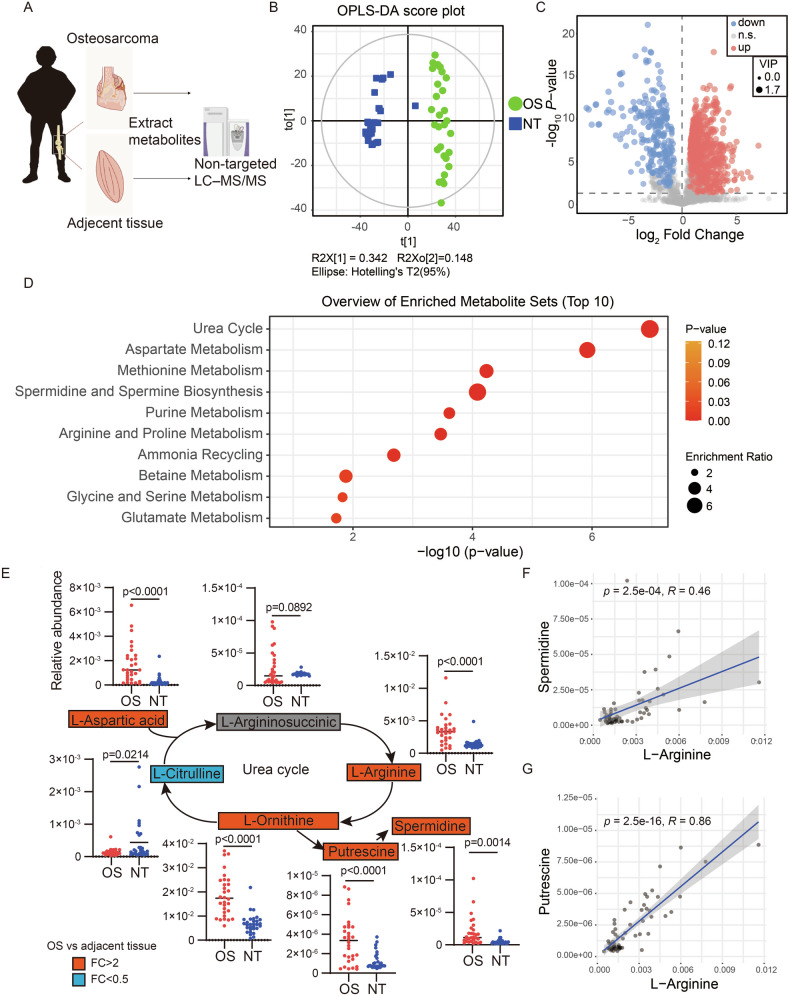
Upregulation of arginine metabolism and polyamine biosynthesis in osteosarcoma. **A** Schematic representation of the experimental design for non-targeted metabolomics to investigate metabolic alterations in osteosarcoma. **B** Score scatter plot from the orthogonal partial least squares discriminant analysis (OPLS-DA) model comparing osteosarcoma tissues (OS, *n* = 30) to normal adjacent tissues (NT, *n* = 30). **C** Analysis of differential metabolites between osteosarcoma (OS) and normal adjacent tissues (NT) derived from non-targeted metabolomics. Statistical analyses were conducted using paired *t*-test. **D** Pathway enrichment analysis based on differential metabolites identified in the non-targeted metabolomics data. **E** Quantitative analysis of metabolites involved in the urea cycle, as determined by non-targeted metabolomics. Data are expressed as mean ± standard deviation (SD). Statistical analyses were conducted using paired *t*-test. **F**, **G** Scatter plots illustrate the Pearson correlation between levels of arginine and polyamines, highlighting their interdependencies in osteosarcoma metabolism. Significance levels are denoted as follows: ***P ≤ 0.001, **P ≤ 0.01, *P ≤ 0.05, n.s. not significant.

To investigate metabolic alterations in osteosarcoma, we performed untargeted metabolomics on both tumor and adjacent non-tumor tissues from 30 osteosarcoma patients (Fig. 1A). Orthogonal projections to latent structures-discriminant analysis (OPLS-DA) revealed a clear metabolic distinction between osteosarcoma and non-tumor tissues, as shown by the OPLS-DA score plot (Fig. 1B). Most samples clustered within the 95% confidence interval of Hotelling’s T-squared ellipse, indicating the robustness of our model.

Using multivariate statistical analysis, we focused on metabolites with high variable importance in the projection (VIP) scores from the OPLS-DA model and *p*-values < 0.05 (Student’s *t*-tests). We identified 20 metabolites that were downregulated and 67 that were upregulated in osteosarcoma tissues (Fig. 1C). Enrichment analysis with MetaboAnalyst 6.0 underscored several significantly altered pathways, including the urea cycle, aspartate metabolism, methionine metabolism, and the biosynthesis of spermidine and spermine, along with arginine and proline metabolism (Fig. 1D).

A comprehensive analysis of these pathways revealed an aberrant increase in arginine levels, driving metabolic flux preferentially toward polyamine synthesis rather than guanidinoacetate production (Fig. 1E). Correlation analyses further demonstrated strong positive associations between arginine, a key substrate for polyamine biosynthesis, and polyamines such as putrescine and spermidine (Fig. 1F, G). Although glutamine is also an important precursor for ornithine and polyamine production [11, 12], its levels were significantly reduced in osteosarcoma relative to non-tumor tissues (Fig. S1A, S1B). Collectively, these findings indicate that enhanced polyamine synthesis is closely linked to arginine level.

### Targeting polyamine synthesis to mitigate osteosarcoma growth

**Fig. 2:**
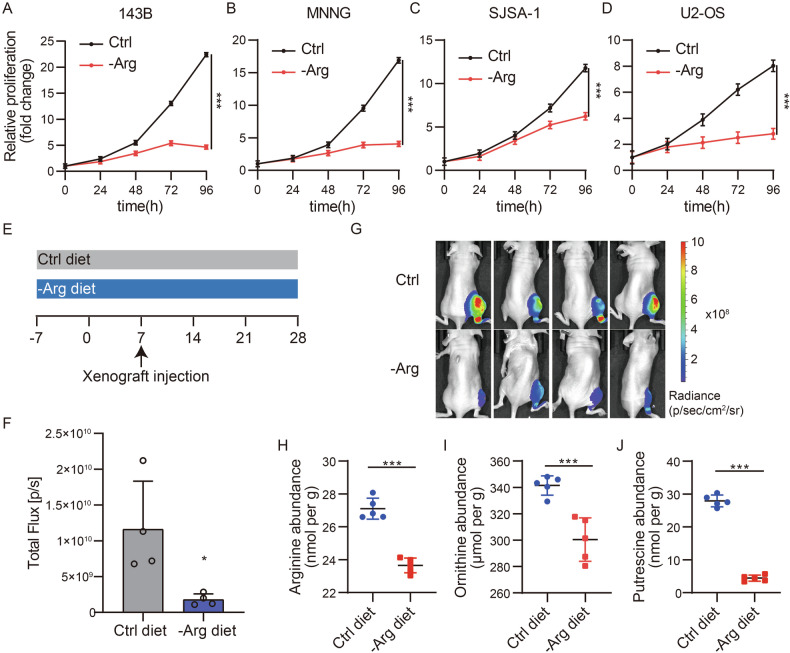
Inhibition of polyamine synthesis suppresses osteosarcoma growth. **A**–**D** Relative growth curves of osteosarcoma cell lines 143B, MNNG, SJSA-1, and U2-OS cultured in normal control medium or under arginine deprivation. Data are expressed as mean ± standard deviation (SD), with each condition replicated three times (*n* = 3). **E** Schematic representation of the experimental setup for evaluating the in vivo growth of 143B-luc under a normal control diet compared to an arginine-deprived diet. **F** Representative images of orthotopic 143B-luc tumors in mice, with four subjects per treatment group (*n* = 4). **G** Quantification of in vivo luciferase signal intensity from 143B-luc tumors, indicating tumor growth dynamics. Data are presented as mean ± SD, with four animals per group (*n* = 4). Statistical analyses were conducted using paired *t*-test. **H**–**J** Measurement of arginine, ornithine, and putrescine concentrations in orthotopic 143B-luc tumors, showcasing metabolic changes associated with diet. Results are expressed as mean ± SD for four subjects per group (*n* = 4). Statistical analyses were conducted using paired *t*-test. Significance levels are denoted as follows: ***P ≤ 0.001, **P ≤ 0.01, *P ≤ 0.05, n.s. not significant.

To assess the role of polyamine in tumor proliferation, we cultured osteosarcoma cell lines in an arginine-depleted medium and monitored cell growth via viability assays over 24, 48, 72, and 96 h. Compared to cells grown in standard DMEM, arginine-deprived cells exhibited markedly reduced proliferation in vitro (Fig. 2A–D), underscoring the essential contribution of polyamine to osteosarcoma cell expansion.

To validate these findings in vivo, we employed an orthotopic 143B xenograft model in which mice were fed either a standard or an arginine-deficient diet (Fig. 2E). Tumor measurements revealed that mice on the arginine-deficient regimen developed significantly smaller tumors than those receiving the standard diet (Fig. 2F, G). Biochemical analyses confirmed notable alterations in arginine, ornithine, and polyamine levels within the tumor microenvironment (Fig. 2H–J). These data indicate that restricting polyamine can be a powerful strategy to suppress osteosarcoma tumor expansion.

### Targeting polyamine synthesis enhances TCR-T cell cytotoxicity against osteosarcoma

**Fig. 3:**
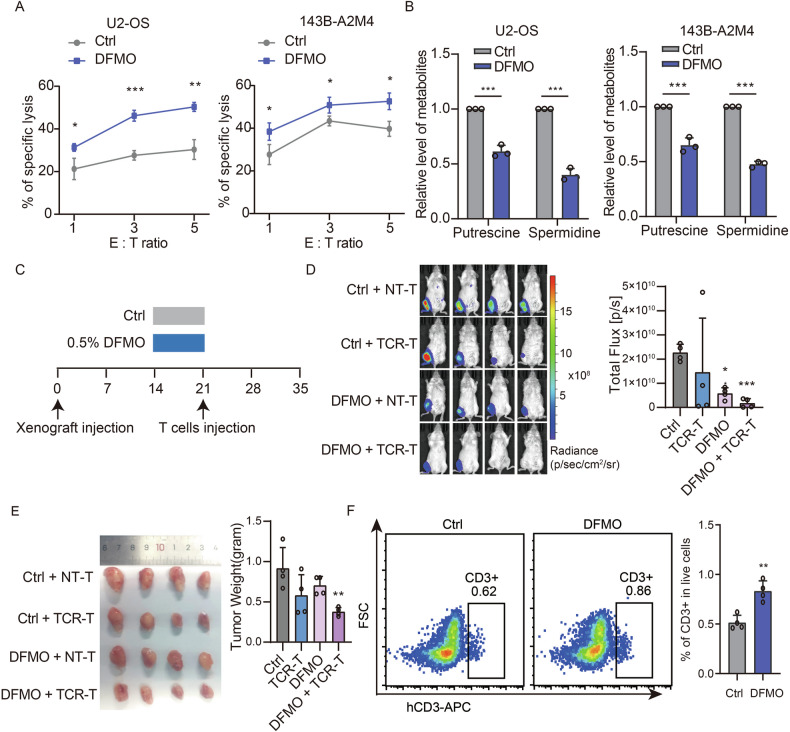
Inhibition of polyamine synthesis enhances TCR-T cell-mediated cytotoxicity in osteosarcoma. **A** TCR-T cell-mediated cytotoxicity against U2-OS and 143B-A2M4 cells at different E:T ratio (effector cells:target cells), with and without DFMO pretreatment (5 mM). Data are expressed as mean ± SD, with three biological replicates per condition (*n* = 3). Statistical significance was assessed using an unpaired *t*-test. B. Levels of putrescine and spermidine in U2-OS and 143B-A2M4 cells following DFMO treatment. Data are presented as mean ± SD, *n* = 3 per group. Statistical analysis was performed using two-way ANOVA. **C** Schematic depiction of the experimental protocol for assessing in vivo TCR-T cell-mediated cytotoxicity of 143B-A2M4-luc cells, with or without DFMO pretreatment (0.5% in drinking water). **D** Representative images and quantification of in vivo luciferase signal intensity from orthotopic 143B-luc tumors treated with transduced (TCR-T) or non-transduced (NT-T) T cells. Data are presented as mean ± SD for four experimental subjects per group (*n* = 4). Statistical evaluation utilized one-way ANOVA. **E** Representative images of orthotopic 143B-A2M4-luc tumors and subsequent tumor weight analysis post-treatment. Data are shown as mean ± SD, with four subjects per treatment group (*n* = 4). Statistical analysis was conducted using one-way ANOVA. **F** Representative images of CD3 staining in 143B-A2M4 orthotopic tumors, gated on live cells, with corresponding quantification analysis. Data are presented as mean ± SD (*n* = 4). Statistical significance was determined using an unpaired *t*-test. Significance levels are indicated as follows: ***P ≤ 0.001, **P ≤ 0.01, *P ≤ 0.05, n.s. not significant.

Previous studies have demonstrated that suppressing polyamine synthesis can increase the effectiveness of immune checkpoint therapies in certain cancers [13, 14]. Building on this insight, we hypothesized that targeting polyamine metabolism could similarly improve osteosarcoma immunogenicity and the efficacy of T-cell–based therapies. To test this, we conducted in vitro cytotoxicity assays with HLA-A*02:01 and MAGEA4 (A2M4) TCR-engineered T cells (TCR-T) against the U2-OS osteosarcoma cell line, which naturally expresses HLA-A*02:01 and MAGEA4. Specifically, we employed T cells engineered to recognize MAGEA4 presented by HLA-A*02:01. Pretreating U2-OS cells with the ODC1 inhibitor difluoromethylornithine (DFMO) significantly enhanced A2M4 TCR-T cell-mediated cytotoxicity, as indicated by LDH release (Figs. 3A and S2). A similar increase in T-cell-induced killing was observed in 143B-A2M4 cells (143B cells engineered to overexpress HLA-A*02:01 and MAGEA4) following DFMO pretreatment (Fig. 3A). Consistent with these results, polyamine measurements in DFMO-treated U2-OS and 143B-A2M4 cultures revealed a substantial decrease in putrescine and spermidine at two days post-DFMO treatment (Fig. 3B).

To evaluate the therapeutic benefit of inhibiting polyamine synthesis in vivo, we used an orthotopic model in immunodeficient NSFG mice implanted intratibially with luciferase-expressing 143B-A2M4 cells (Fig. 3C). After two weeks of tumor growth, mice received either a 0.5% DFMO solution or normal water for seven days, followed by a single intravenous injection of A2M4 TCR-T cells at the end of the third week. Imaging and tumor analyses during the fifth week revealed that while TCR-T cell therapy alone modestly attenuated tumor growth, this effect was significantly enhanced by short-term DFMO pretreatment (Fig. 3D, E). Moreover, we observed increased T-cell infiltration in DFMO-treated tumors (Fig. 3F). These data highlight the potential of polyamine synthesis inhibition not only to impede osteosarcoma progression but also to bolster the immune microenvironment, thereby improving T-cell-mediated cytotoxicity.

### Impact of AZIN1-mediated polyamine synthesis on osteosarcoma progression

**Fig. 4:**
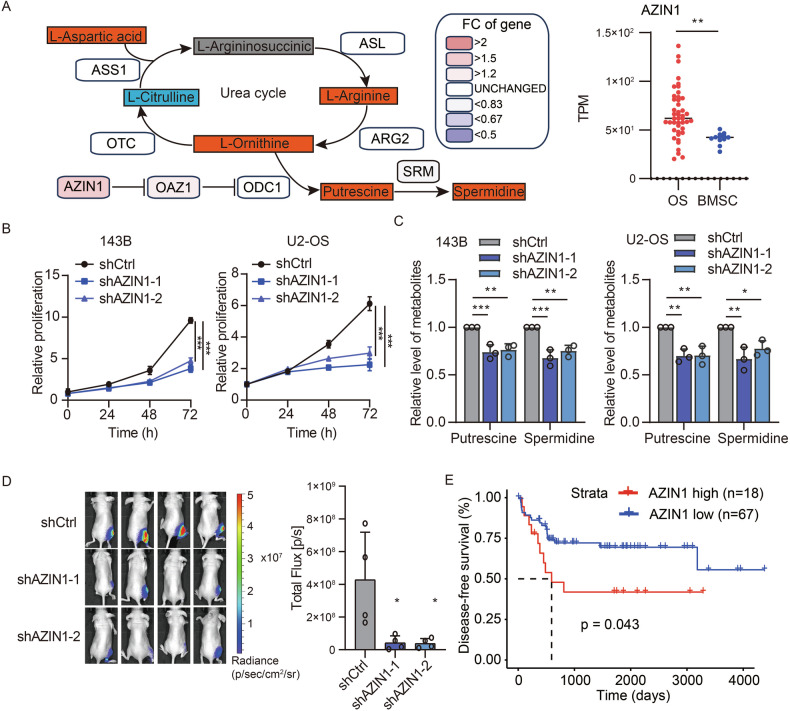
AZIN1 is critical for polyamine production and cell proliferation in osteosarcoma. **A** Schematic representation integrating non-targeted metabolomics and RNA-seq data to show the expression of arginine metabolism-related genes in osteosarcoma and bone marrow stromal cells (BMSC) using publicly available datasets (GSE87624 and E-MTAB-7925). Data are presented as mean ± SD, with sample sizes of *n* = 12 for BMSC and *n* = 44 for OS. Statistical analysis was performed using an unpaired *t*-test. **B** Relative in vitro growth curves of 143B and U2-OS osteosarcoma cell lines with or without *AZIN1* knockdown. Data are expressed as mean ± SD, *n* = 3. Statistical analysis utilized one-way ANOVA. **C** Levels of putrescine and spermidine in 143B and U2-OS cells following *AZIN1* knockdown. Data are shown as mean ± SD, with three replicates per group (*n* = 3). Two-way ANOVA was used for statistical analysis. **D** Representative images and quantification of in vivo luciferase signal intensity in orthotopic 143B-luc tumors with or without *AZIN1* knockdown. Data are presented as mean ± SD, *n* = 4 per group. Statistical evaluation was conducted using one-way ANOVA. **E** Kaplan-Meier survival analysis comparing patients stratified by high (*n* = 18) and low (*n* = 67) AZIN1 expression levels from the TARGET_OS dataset. Statistical evaluation was conducted using the log-rank test. Significance levels are indicated as follows: ***P ≤ 0.001, **P ≤ 0.01, *P ≤ 0.05, n.s. not significant.

To elucidate the mechanisms underlying aberrant polyamine synthesis in osteosarcoma, we examined transcriptional profiles of key genes in the arginine–polyamine metabolic pathway using publicly available RNA-seq data. Although the expression levels of ARG2 and ODC1, an essential enzyme for polyamine synthesis [7], were comparable to those in normal tissues, we detected a marked upregulation of AZIN1 (Figs. 4A and S3). Given AZIN1’s known role in regulating ODC1 stability and activity [15], we hypothesized that AZIN1 overexpression underlies the dysregulated arginine-polyamine metabolism characteristic of osteosarcoma.

To test this hypothesis, we generated 143B and U2-OS osteosarcoma cell lines with stable *AZIN1* knockdown and conducted cell viability assays. Both cell lines displayed substantially reduced proliferation following *AZIN1* knockdown (Figs. 4B and S4). Consistent with AZIN1’s regulatory role, we observed a significant decrease in intracellular polyamine levels in these knockdown cells (Fig. 4C). Further supporting these findings, an orthotopic tumor model in mice revealed that *AZIN1* knockdown markedly inhibited tumor growth in vivo (Fig. 4D). Clinically, Kaplan-Meier analysis of patient data indicated that high AZIN1 expression correlates with significantly shorter disease-free survival compared to low expression (Fig. 4E). Taken together, these results highlight the pivotal function of AZIN1 in osteosarcoma proliferation and underscore its potential as a therapeutic target.

### AZIN1 regulation of polyamine synthesis modulates cell cycle dynamics in osteosarcoma

**Fig. 5:**
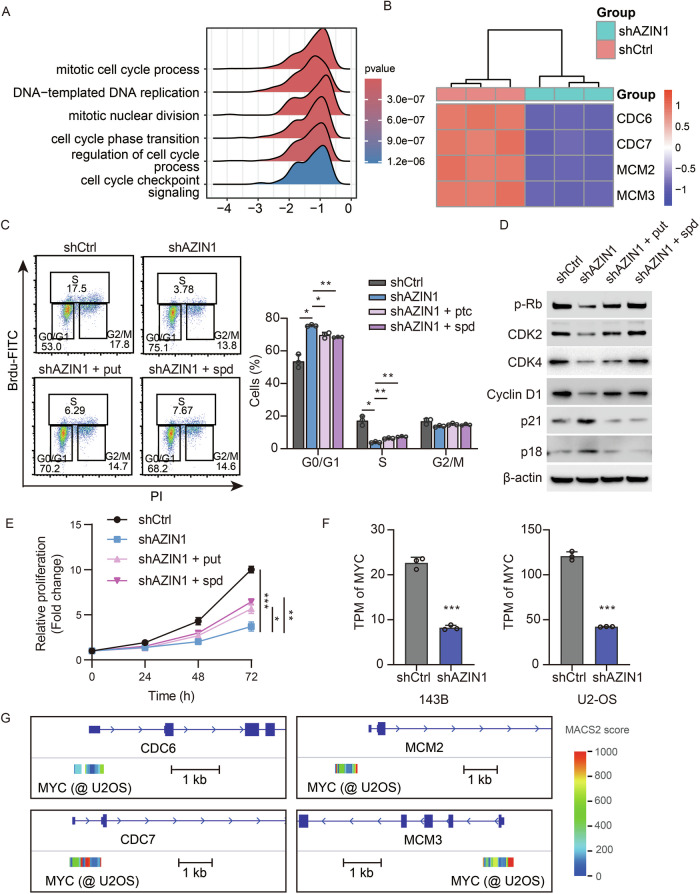
AZIN1 facilitates cell cycle progression in osteosarcoma cells in a polyamine-dependent manner. **A** Representative gene set enrichment analysis (GSEA) results from RNA-seq data of 143B cells with or without *AZIN1* knockdown, highlighting changes in cell cycle-related gene expression. **B** Gene expression heatmap displaying the transcriptional levels of cell cycle-regulating genes in 143B cells, comparing conditions with and without *AZIN1* knockdown. **C** Representative flow cytometry plots of cell cycle analysis in 143B cells following *AZIN1* knockdown and supplementation with 10 μM putrescine and 5 μM spermidine, assessed by BrdU pulse-labeling and PI staining. Quantification of cell cycle phase distribution is on the right. Data are mean ± SD (*n* = 3). Statistical significance was determined using one-way ANOVA. **D** Western blot analysis of cell cycle regulators in 143B cells with AZIN1 knockdown, supplemented with putrescine and spermidine. **E** In vitro growth curves of 143B cells with *AZIN1* knockdown and subsequent supplementation with putrescine and spermidine. Data are mean ± SD (*n* = 3). Statistical analysis was performed using one-way ANOVA. **F** Normalized expression levels of MYC from RNA-seq data of 143B and U2-OS cells with and without *AZIN1* knockdown. Data are mean ± SD (*n* = 3). Statistical significance was assessed using an unpaired *t*-test. **G** Representative IGV tracks showing MYC ChIP-seq binding on promoters of cell cycle-regulating genes in U2-OS cells, derived from the ChIP-Atlas database. Significance levels are indicated as follows: ***P ≤ 0.001, **P ≤ 0.01, *P ≤ 0.05, n.s. not significant.

To investigate how AZIN1 influences osteosarcoma cell proliferation, we performed RNA sequencing on 143B, and U2-OS cells stably knocked down for *AZIN1* and compared the results to control cells. Gene Set Enrichment Analysis (GSEA) revealed marked downregulation of gene sets tied to cell proliferation, particularly those associated with the cell cycle, nuclear division, and DNA replication (Figs. 5A and S5A). Correspondingly, crucial cell cycle regulatory genes, including CDC6, CDC7, MCM2, and MCM3 [16–18], were substantially suppressed following *AZIN1* knockdown (Figs. 5B and S5B). A similar expression pattern emerged in 143B cells cultured in an arginine-depleted medium, and flow cytometry also showed that the cell cycle was infected (Fig. S5C, D).

Given the established function of polyamines in cell cycle regulation, we hypothesized that AZIN1 promotes the osteosarcoma cell cycle by modulating ODC1 activity and, consequently, polyamine synthesis. Flow cytometric analysis of *AZIN1*-knockdown 143B and U2-OS cells revealed an increased proportion of cells in G_0_/G_1_ and a concomitant decrease in S phase, indicative of cell cycle arrest. Supplementing these cells with putrescine or spermidine partly mitigated the G_0_/G_1_ arrest (Figs. 5C and S5E). Consistent with this finding, AZIN1 knockdown led to reduced levels of phosphorylated retinoblastoma protein (p-Rb), CDK2, CDK4, and Cyclin D1, alongside elevated levels of the cyclin-dependent kinase inhibitors p18 and p21. Notably, these molecular changes were partly reversed by the addition of putrescine or spermidine (Figs. 5D and S5F). Restoring polyamine levels in *AZIN1*-knockdown cells further led to a resurgence in proliferation in both 143B and U2-OS cell lines (Figs. 5E and S5G).

Extending these observations, we found that either *AZIN1* knockdown or inhibition of polyamine synthesis diminished MYC expression (Fig. 5F). Furthermore, chromatin immunoprecipitation and promoter analyses demonstrated that MYC may serve as a regulatory hub for key cell cycle genes (Fig. 5G). Collectively, these results highlight the pivotal role of the AZIN1-polyamine-MYC axis in governing cell cycle progression in osteosarcoma.

### Impairment of osteosarcoma immunogenicity by AZIN1

**Fig. 6:**
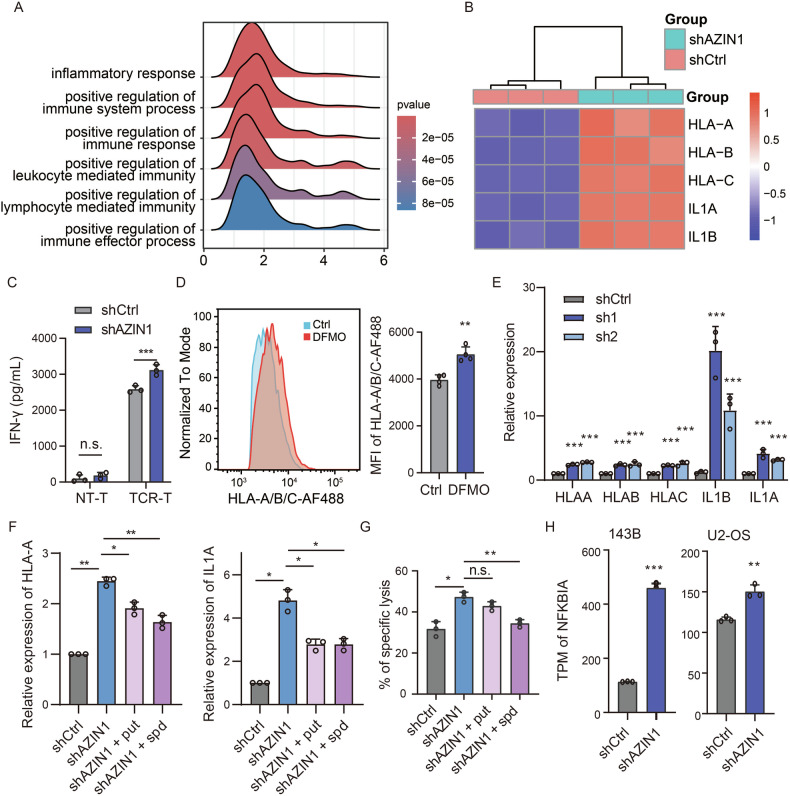
AZIN1-mediated polyamine production alters osteosarcoma immunogenicity and enhances T-cell-mediated cytotoxicity. **A** Gene set enrichment analysis (GSEA) of RNA-seq data from 143B cells, showing changes in immune response-related gene expression with and without *AZIN1* knockdown. **B** Heatmap of transcriptional levels of genes regulating T cell responses in 143B cells, under conditions with and without *AZIN1* knockdown. **C** ELISA quantification of IFN-γ levels in T cell co-cultures with 143B-A2M4 cells, comparing AZIN1 knockdown with control conditions. Data are expressed as mean ± SD (*n* = 3). Statistical evaluation was performed using one-way ANOVA. **D** Representative image of HLA-A/B/C staining in 143B-A2M4 orthotopic tumors, gated on live and HLA-A/B/C positive cells, with mean fluorescence intensity (MFI) quantification analysis. Data are presented as mean ± SD (*n* = 4). Statistical significance was determined using an unpaired *t*-test. **E** Quantitative PCR (qPCR) analysis of T cell activation-related gene expression in 143B cells with and without *AZIN1* knockdown. Data are expressed as mean ± SD, *n* = 3. Statistical analysis was performed using two-way ANOVA. **F** qPCR analysis of HLA-A and IL1A expression levels in 143B cells post-*AZIN1* knockdown and supplemented with either putrescine (put) or spermidine (spd). Data are expressed as mean ± SD, *n* = 3. Statistical analysis was conducted using one-way ANOVA. **G** Assessment of TCR-T cell-mediated cytotoxicity against 143B-A2M4 cells with *AZIN1* knockdown and supplementation with 10 μM putrescine (put) or 5 μM spermidine (spd). Data are presented as mean ± SD, *n* = 3. Statistical evaluation utilized one-way ANOVA. **H** Normalized expression levels of NFKBIA in RNA-seq data from 143B and U2-OS cells under conditions with and without *AZIN1* knockdown. Data are presented as mean ± SD (*n* = 3). Statistical significance was assessed using an unpaired *t*-test. Significance levels are denoted as follows: ***P ≤ 0.001, **P ≤ 0.01, *P ≤ 0.05, n.s. not significant.

We performed a comprehensive analysis of RNA-seq data from 143B and U2-OS osteosarcoma cells with AZIN1 knockdown to investigate how polyamine metabolism modulates the osteosarcoma immune microenvironment. Gene set enrichment analysis (GSEA) revealed marked upregulation of immune-related gene sets, including inflammatory responses, leukocyte proliferation, migration, activation, and T-cell-mediated immunity, following *AZIN1* knockdown (Figs. 6A and S6A). Further examination showed increased expression of key T-cell activation markers HLA-A/B/C, IL1A, and IL1B (Figs. 6B and S6B). Accordingly, A2M4 TCR-T cells co-cultured with *AZIN1*-knockdown osteosarcoma cells secreted significantly more IFN-γ than those co-cultured with shControl cells (Figs. 6C and S6C). In parallel, flow cytometric analysis of DFMO-treated tumors demonstrated elevated MHC-I (HLA-A/B/C) expression (Fig. 6D).

To validate these observations, we conducted quantitative PCR (qPCR) on *AZIN1*-knockdown U2-OS and 143B cells, confirming the upregulation of HLA-A/B/C, IL1A, and IL1B [19] (Figs. 6E and S6D). ChIP-seq analyses further revealed that MYC may serve as a regulatory hub for these immune-related genes (Fig. S6E).

We next tested whether these effects were dependent on polyamine levels. Supplementation of AZIN1-knockdown cells with putrescine or spermidine partially reversed the upregulation of immune-related genes (Figs. 6F and S6F). Concordantly, TCR-T cytotoxicity assays showed that exogenous polyamine supplementation mitigated the enhanced T-cell-mediated killing of *AZIN1*-knockdown 143B-A2M4 and U2-OS cells (Figs. 6G and S6G). Interestingly, *AZIN1* knockdown also triggered a polyamine-dependent increase in PD-L1 expression (Fig. S7A). Nonetheless, the elevated T-cell cytotoxicity observed after *AZIN1* knockdown was further potentiated by anti-PD-L1 treatment relative to an isotype control (Fig. S7B).

Lastly, to explore the underlying mechanism, we reanalyzed RNA-seq data from 143B and U2-OS cells subjected to *AZIN1* knockdown. Both cell lines showed significantly increased expression of *NFKBIA* (Fig. 6H), which encodes IκBα, a negative feedback regulator preventing NF-κB overactivation [20]. Taken together, these findings highlight the critical role of AZIN1-driven polyamine synthesis in modulating the immune microenvironment, which in turn influences the immunogenicity of osteosarcoma and its vulnerability to T-cell-mediated cytotoxicity.

## Discussion

**Fig. 7:**
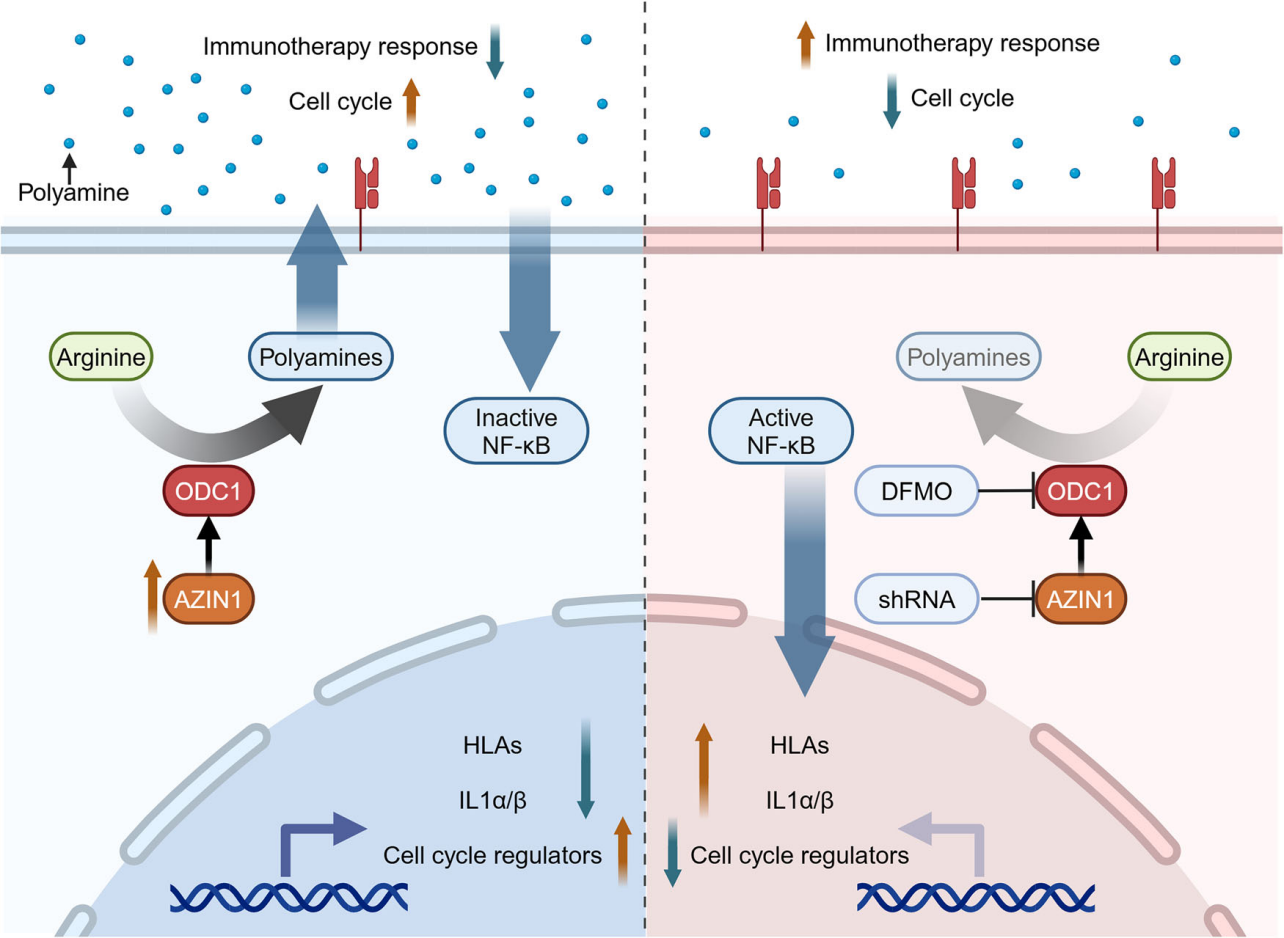
Schematic representation of the proposed hypothesis: elevated AZIN1-driven polyamine production promotes osteosarcoma proliferation and impairs immunotherapy efficacy. In osteosarcoma cells, increased levels of arginine and AZIN1 upregulate the metabolism of arginine into polyamines through AZIN1-mediated activation of ornithine decarboxylase 1 (ODC1). This enhancement in polyamine production supports tumor cell proliferation and impedes the efficacy of immunotherapy. Conversely, when polyamine synthesis is inhibited—either by AZIN1 knockdown or through difluoromethylornithine (DFMO)-mediated inhibition of ODC1-polyamine levels decrease, leading to cell cycle arrest and the upregulation of human leukocyte antigen (HLA) expression and T cell-activating cytokines. These changes augment the cytotoxic efficacy of TCR-engineered T cells against osteosarcoma cells.

Our comprehensive metabolomic profiling provides compelling evidence that osteosarcoma exhibits significant activation of the polyamine synthesis pathway. This upregulation is primarily driven by the overexpression of AZIN1, a critical regulatory protein that governs polyamine biosynthesis. Notably, the knockdown of AZIN1 effectively curtailed tumor growth and heightened the tumor’s sensitivity to immunotherapy (Fig. 7). These findings suggest that osteosarcoma’s characteristic “cold” immune phenotype [21] may, at least in part, stem from dysregulated polyamine metabolism and that targeting this pathway could improve immunotherapeutic outcomes in this challenging cancer.

Our metabolomic analysis offers deeper insights into the metabolic reprogramming in osteosarcoma, particularly highlighting a strong preference for the arginine-driven pathway in polyamine production. We detected markedly elevated levels of arginine, ornithine, and putrescine, accompanied by a notable decrease in glutamine. While polyamines can be synthesized via both arginine- and glutamine-derived pathways [22], our data suggest that osteosarcoma cells predominantly rely on the arginine route. Nonetheless, future studies employing isotope-labeled metabolite tracing will be crucial to validate and further solidify this conclusion.

Polyamines have long been recognized as critical mediators of tumorigenesis and immune modulation, owing to their essential role in promoting cell proliferation and survival [7, 8]. This aligns with our observation of elevated putrescine and spermidine in osteosarcoma tissues. Mechanistically, the AZIN1–polyamine axis emerges as particularly pivotal: increased polyamine availability has been reported to influence chromatin accessibility at the MYC promoter or its regulatory elements, potentially enhancing MYC transcription [23–25]. In turn, MYC is known to regulate genes involved in cell cycle progression [26–28] and may influence immunosuppressive molecules and cytokines [29, 30], which could contribute to tumor growth and immune evasion.

Our findings further highlight the central role of MYC in orchestrating polyamine-mediated proliferation and survival in osteosarcoma. Knockdown of AZIN1 diminishes intracellular MYC levels, leading to reduced expression of cyclins and cyclin-dependent kinases and, consequently, curbing cell cycle progression. In parallel, lower MYC activity may attenuate anti-apoptotic gene expression [31], thereby restraining tumor growth. Notably, polyamine supplementation can restore these tumorigenic features that were lost upon AZIN1 knockdown, underscoring the tight functional interplay between polyamines and MYC in osteosarcoma pathophysiology. These observations support a potential role for MYC within the polyamine-mediated network of osteosarcoma, and highlight it as a candidate for future therapeutic exploration.

Beyond fueling proliferation, polyamines also contribute to immune evasion in osteosarcoma. By shaping immunosuppressive populations, such as regulatory T cells, tumor-associated macrophages (TAMs), myeloid-derived suppressor cells (MDSCs), and dendritic cells (DCs), excess polyamines create a tumor microenvironment that impairs effective antitumor immunity [8, 32]. Mechanistically, the AZIN1-mediated surge in polyamines is associated with altered cytokine and HLA expression, possibly involving MYC-dependent pathways, ultimately skewing local cytokine profiles and reducing immune cell activation. This suite of changes underlies osteosarcoma’s characteristically poor immunogenicity, highlighting how metabolic reprogramming can intersect with immune dysregulation to promote tumor progression.

In light of these insights, targeting the AZIN1-polyamine axis presents a promising strategy for combating osteosarcoma. Given MYC’s downstream involvement, interventions that modulate MYC activity may also hold therapeutic value, although further studies are needed to validate its role in immune modulation. Future research should focus on refining pharmacologic inhibitors of polyamine biosynthesis, determining optimal dosing regimens, and investigating potential synergies between these agents and established or emerging immunotherapies.

## Supplementary information

Uncropped images for WB figures

Supplementary Figures and Tables

Supplementary Table3

## Data Availability

The transcriptome sequencing (mRNA-seq) of 143B and U2-OS cells data generated during the current study have been deposited in the Genome Sequence Archive at National Genomics Data Center, China National Center for Bioinformation / Beijing Institute of Genomics, Chinese Academy of Sciences (GSA-Human: HRA010470) that are publicly accessible at https://ngdc.cncb.ac.cn/gsa-human. All data necessary to support the conclusions are included in the main text and/or Supplementary Materials, with additional data available from the authors upon request.

## References

[1] Moore DD, Luu HH. Osteosarcoma. Cancer Treat Res. 2014;162:65–92. 25070231 10.1007/978-3-319-07323-1_4

[2] Ottaviani G, Jaffe N. The epidemiology of osteosarcoma. Cancer Treat Res. 2009;152:3–13. 20213383 10.1007/978-1-4419-0284-9_1

[3] Yu S, Yao X. Advances on immunotherapy for osteosarcoma. Mol Cancer. 2024;23:192. 39245737 10.1186/s12943-024-02105-9 PMC11382402

[4] Tian H, Cao J, Li B, Nice EC, Mao H, Zhang Y, et al. Managing the immune microenvironment of osteosarcoma: the outlook for osteosarcoma treatment. Bone Res. 2023;11:11. 36849442 10.1038/s41413-023-00246-z PMC9971189

[5] Chapman NM, Chi H. Metabolic rewiring and communication in cancer immunity. Cell Chem Biol. 2024;31:862–83. 38428418 10.1016/j.chembiol.2024.02.001 PMC11177544

[6] Richardson LG, Miller JJ, Kitagawa Y, Wakimoto H, Choi BD, Curry WT. Implications of idh mutations on immunotherapeutic strategies for malignant glioma. Neurosurg Focus. 2022;52:E6. 35104795 10.3171/2021.11.FOCUS21604

[7] Pegg AE. Functions of polyamines in mammals. J Biol Chem. 2016;291(Jul):14904–12. 27268251 10.1074/jbc.R116.731661 PMC4946908

[8] Hesterberg RS, Cleveland JL, Epling-Burnette PK. Role of polyamines in immune cell functions. Med Sci (Basel). 2018;6:22. 29517999 10.3390/medsci6010022 PMC5872179

[9] Shantz LM, Levin VA. Regulation of ornithine decarboxylase during oncogenic transformation: mechanisms and therapeutic potential. Amino Acids. 2007;33:213–23. 17443268 10.1007/s00726-007-0531-2

[10] Ramos-Molina B, Lambertos A, Penafiel R. Antizyme inhibitors in polyamine metabolism and beyond: physiopathological implications. Med Sci (Basel). 2018;6:89. 30304856 10.3390/medsci6040089 PMC6313458

[11] Puleston DJ, Villa M, Pearce EL. Ancillary activity: beyond core metabolism in immune cells. Cell Metab. 2017;26:131–41. 28683280 10.1016/j.cmet.2017.06.019 PMC5546226

[12] Wu R, Chen X, Kang S, Wang T, Gnanaprakasam JR, Yao Y, et al. De novo synthesis and salvage pathway coordinately regulate polyamine homeostasis and determine T cell proliferation and function. Sci Adv. 2020;6:eabc4275. 33328226 10.1126/sciadv.abc4275 PMC7744078

[13] Lian J, Liang Y, Zhang H, Lan M, Ye Z, Lin B, et al. The role of polyamine metabolism in remodeling immune responses and blocking therapy within the tumor immune microenvironment. Front Immunol. 2022;13:912279. 36119047 10.3389/fimmu.2022.912279 PMC9479087

[14] Alexander ET, Mariner K, Donnelly J, Phanstiel OT, Gilmour SK. Polyamine blocking therapy decreases survival of tumor-infiltrating immunosuppressive myeloid cells and enhances the antitumor efficacy of pd-1 blockade. Mol Cancer Ther. 2020;19:2012–22. 32747421 10.1158/1535-7163.MCT-19-1116 PMC7541445

[15] Tulluri V, Nemmara VV. Role of antizyme inhibitor proteins in cancers and beyond. Onco Targets Ther. 2021;14:667–82. 33531815 10.2147/OTT.S281157 PMC7846877

[16] Sun Y, Cheng Z, Liu S. Mcm2 in human cancer: functions, mechanisms, and clinical significance. Mol Med. 2022;28:128. 36303105 10.1186/s10020-022-00555-9 PMC9615236

[17] Suski JM, Ratnayeke N, Braun M, Zhang T, Strmiska V, Michowski W, et al. Cdc7-independent g1/s transition revealed by targeted protein degradation. Nature. 2022;605:357–65. 35508654 10.1038/s41586-022-04698-x PMC9106935

[18] Pellarin I, Dall’Acqua A, Favero A, Segatto I, Rossi V, Crestan N, et al. Cyclin-dependent protein kinases and cell cycle regulation in biology and disease. Signal Transduct Target Ther. 2025;10:11. 39800748 10.1038/s41392-024-02080-z PMC11734941

[19] Van Den Eeckhout B, Tavernier J, Gerlo S. Interleukin-1 as innate mediator of t cell immunity. Front Immunol. 2020;11:621931. 33584721 10.3389/fimmu.2020.621931 PMC7873566

[20] Kolesnichenko M, Mikuda N, Hopken UE, Kargel E, Uyar B, Tufan AB, et al. Transcriptional repression of nfkbia triggers constitutive ikk- and proteasome-independent p65/rela activation in senescence. Embo J. 2021;40:e104296. 33459422 10.15252/embj.2019104296 PMC7957429

[21] Bonaventura P, Shekarian T, Alcazer V, Valladeau-Guilemond J, Valsesia-Wittmann S, Amigorena S, et al. Cold tumors: a therapeutic challenge for immunotherapy. Front Immunol. 2019;10:168. 30800125 10.3389/fimmu.2019.00168 PMC6376112

[22] Wang X, Ying W, Dunlap KA, Lin G, Satterfield MC, Burghardt RC, et al. Arginine decarboxylase and agmatinase: an alternative pathway for de novo biosynthesis of polyamines for development of mammalian conceptuses. Biol Reprod. 2014;90:84. 24648395 10.1095/biolreprod.113.114637

[23] Coni S, Bordone R, Ivy DM, Yurtsever ZN, Di Magno L, D’Amico R, et al. Combined inhibition of polyamine metabolism and eif5a hypusination suppresses colorectal cancer growth through a converging effect on myc translation. Cancer Lett. 2023;559:216120. 36893894 10.1016/j.canlet.2023.216120

[24] Coni S, Serrao SM, Yurtsever ZN, Di Magno L, Bordone R, Bertani C, et al. Blockade of eif5a hypusination limits colorectal cancer growth by inhibiting myc elongation. Cell Death Dis. 2020;11:1045. 33303756 10.1038/s41419-020-03174-6 PMC7729396

[25] Kumar N, Basundra R, Maiti S. Elevated polyamines induce c-myc overexpression by perturbing quadruplex-wc duplex equilibrium. Nucleic Acids Res. 2009;37:3321–31. 19324889 10.1093/nar/gkp196 PMC2691834

[26] Liu C, Kudo T, Ye X, Gascoigne K. Cell-to-cell variability in myc dynamics drives transcriptional heterogeneity in cancer cells. Cell Rep. 2023;42:112401. 37060565 10.1016/j.celrep.2023.112401

[27] Garcia-Gutierrez L, Bretones G, Molina E, Arechaga I, Symonds C, Acosta JC, et al. Myc stimulates cell cycle progression through the activation of cdk1 and phosphorylation of p27. Sci Rep. 2019;9:18693. 31822694 10.1038/s41598-019-54917-1 PMC6904551

[28] Bretones G, Delgado MD, Leon J. Myc and cell cycle control. Biochim Biophys Acta. 2015;1849:506–16. 24704206 10.1016/j.bbagrm.2014.03.013

[29] Krenz B, Lee J, Kannan T, Eilers M. Immune evasion: an imperative and consequence of myc deregulation. Mol Oncol. 2024;18:2338–55. 38957016 10.1002/1878-0261.13695 PMC11459038

[30] Wu X, Nelson M, Basu M, Srinivasan P, Lazarski C, Zhang P, et al. Myc oncogene is associated with suppression of tumor immunity and targeting myc induces tumor cell immunogenicity for therapeutic whole cell vaccination. J Immunother Cancer. 2021;9:e1388. 10.1136/jitc-2020-001388 PMC7993333 33757986

[31] Llombart V, Mansour MR. Therapeutic targeting of “undruggable” myc. Ebiomedicine. 2022;75:103756. 34942444 10.1016/j.ebiom.2021.103756 PMC8713111

[32] Holbert CE, Casero RJ, Stewart TM. Polyamines: the pivotal amines in influencing the tumor microenvironment. Discov Oncol. 2024;15:173. 38761252 10.1007/s12672-024-01034-9 PMC11102423

