# Supplementary material for: AZIN1-dependent polyamine synthesis accelerates tumor cell cycle progression and impairs effector T-cell function in osteosarcoma
Source: Cell Death Dis. 2025 Apr 17;16(1):310. doi: 10.1038/s41419-025-07640-x (PMC12006533; doi:10.1038/s41419-025-07640-x)
Supplement: Supplementary file 1 — Uncropped images for WB figures [file 41419_2025_7640_MOESM1_ESM.pptx]

## Slide 1
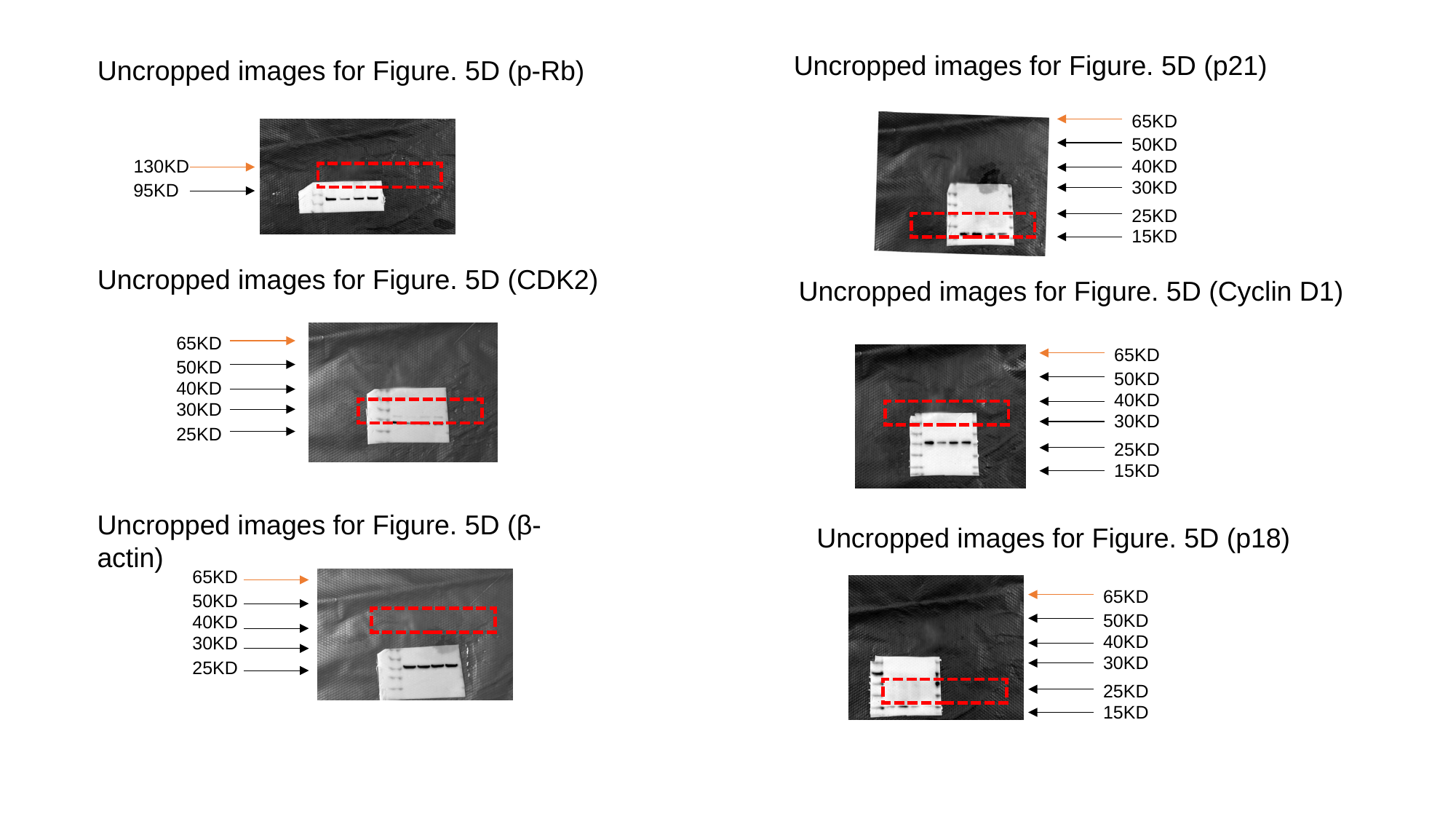

Uncropped images for Figure. 5D (p21)
Uncropped images for Figure. 5D (p-Rb)
65KD
50KD
40KD
30KD
25KD
15KD
130KD
95KD
Uncropped images for Figure. 5D (CDK2)
Uncropped images for Figure. 5D (Cyclin D1)
65KD
50KD
40KD
30KD
25KD
65KD
50KD
40KD
30KD
25KD
15KD
Uncropped images for Figure. 5D (β-actin)
Uncropped images for Figure. 5D (p18)
65KD
50KD
40KD
30KD
25KD
65KD
50KD
40KD
30KD
25KD
15KD

## Slide 2
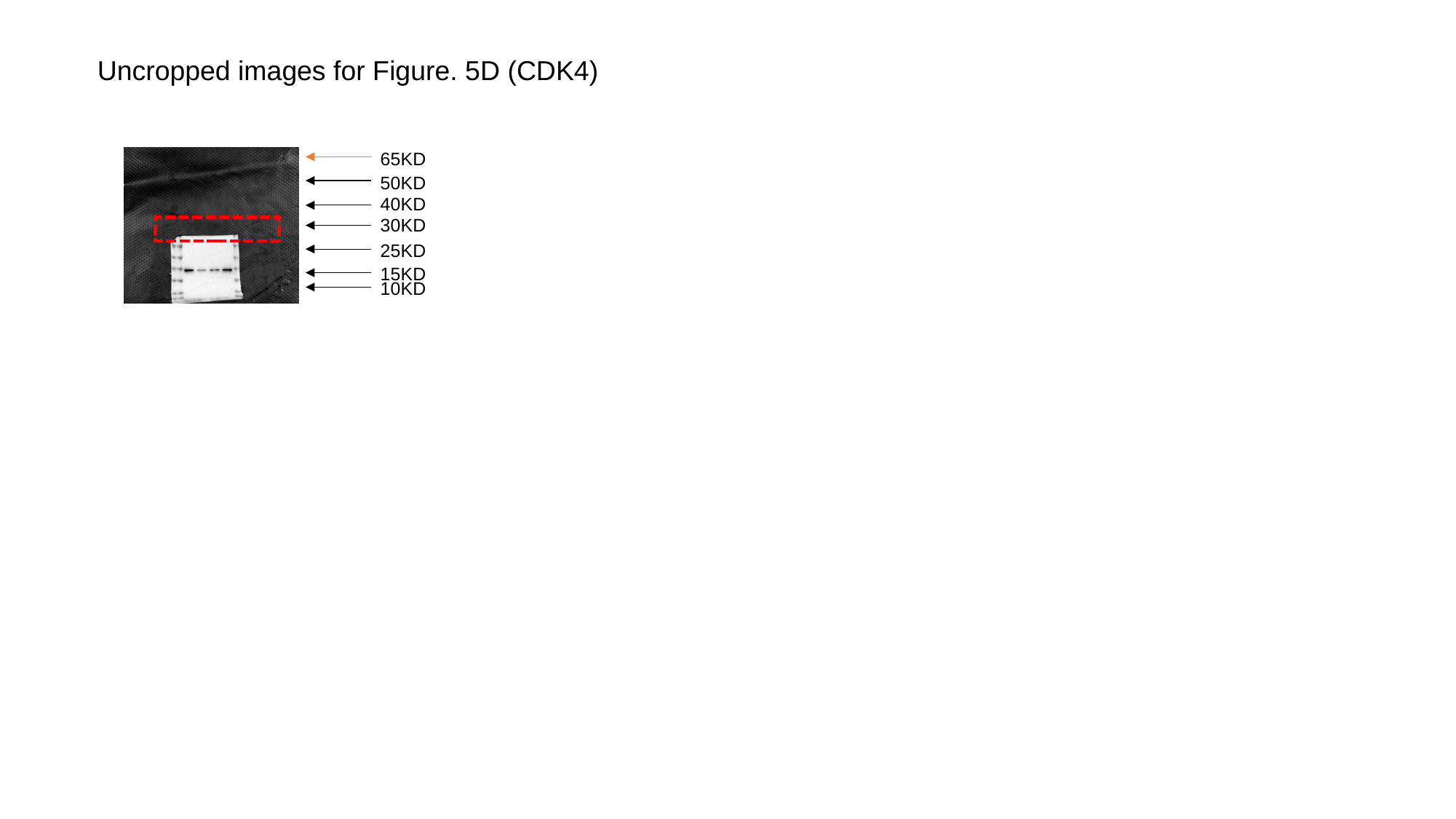

Uncropped images for Figure. 5D (CDK4)
65KD
50KD
40KD
30KD
25KD
15KD
10KD

## Slide 3
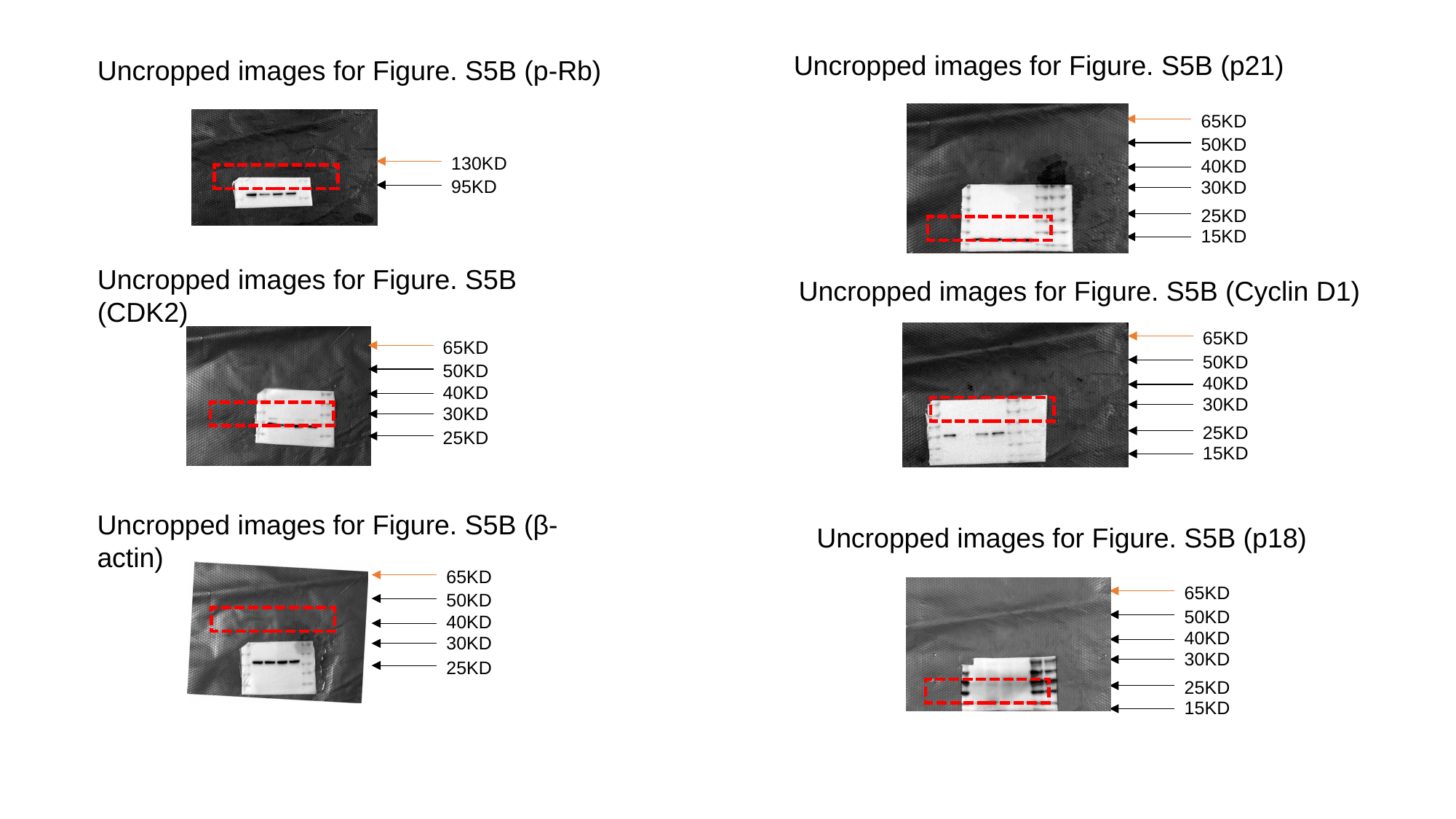

Uncropped images for Figure. S5B (p21)
Uncropped images for Figure. S5B (p-Rb)
65KD
50KD
40KD
30KD
25KD
15KD
130KD
95KD
Uncropped images for Figure. S5B (CDK2)
Uncropped images for Figure. S5B (Cyclin D1)
65KD
50KD
40KD
30KD
25KD
15KD
65KD
50KD
40KD
30KD
25KD
Uncropped images for Figure. S5B (β-actin)
Uncropped images for Figure. S5B (p18)
65KD
50KD
40KD
30KD
25KD
65KD
50KD
40KD
30KD
25KD
15KD

## Slide 4
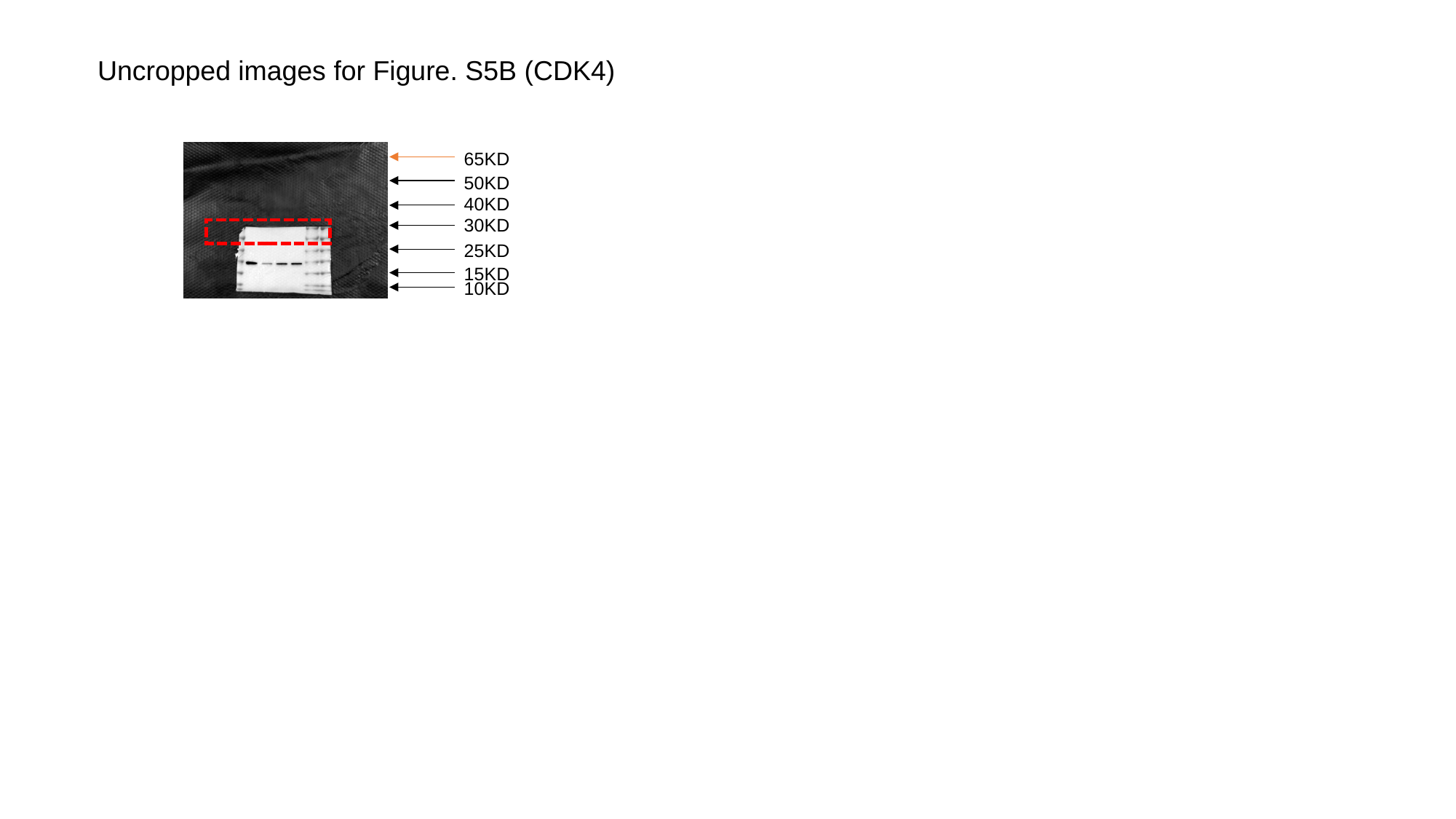

Uncropped images for Figure. S5B (CDK4)
65KD
50KD
40KD
30KD
25KD
15KD
10KD

## Slide 5
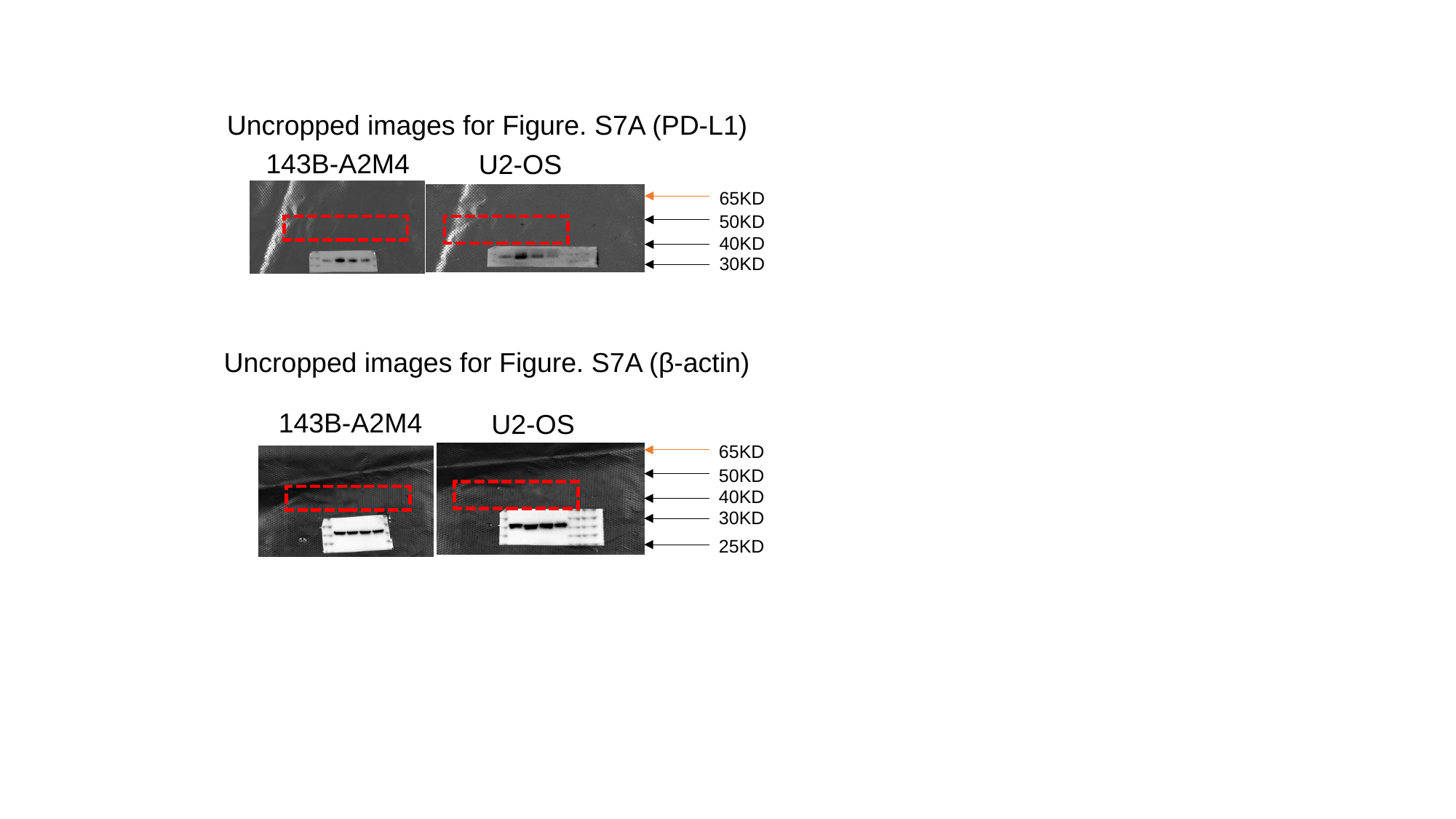

Uncropped images for Figure. S7A (PD-L1)
143B-A2M4
U2-OS
65KD
50KD
40KD
30KD
Uncropped images for Figure. S7A (β-actin)
143B-A2M4
U2-OS
65KD
50KD
40KD
30KD
25KD
