# Supplementary material for: AZIN1-dependent polyamine synthesis accelerates tumor cell cycle progression and impairs effector T-cell function in osteosarcoma
Source: Cell Death Dis. 2025 Apr 17;16(1):310. doi: 10.1038/s41419-025-07640-x (PMC12006533; doi:10.1038/s41419-025-07640-x)
Supplement: Supplementary file 2 — Supplementary Figures and Tables [file 41419_2025_7640_MOESM2_ESM.docx]

**AZIN1-dependent Polyamine Synthesis Accelerates Tumor Cell Cycle Progression and Impairs Effector T-Cell Function in Osteosarcoma**

Jiaming Yu, Chuanxia Zhang, Qinkai Zhang, Bing Lu, Guohao Lu, Chunxiao Zhang, Ru Qiu, Xinyue Wang, Changye Zou, Junjun Chu, Haizhou Li, and Wei Zhao

**Contents**

**Supplemental Figure 1-7**

**Supplemental Table 1-2**

**Supplemental Figure 1**


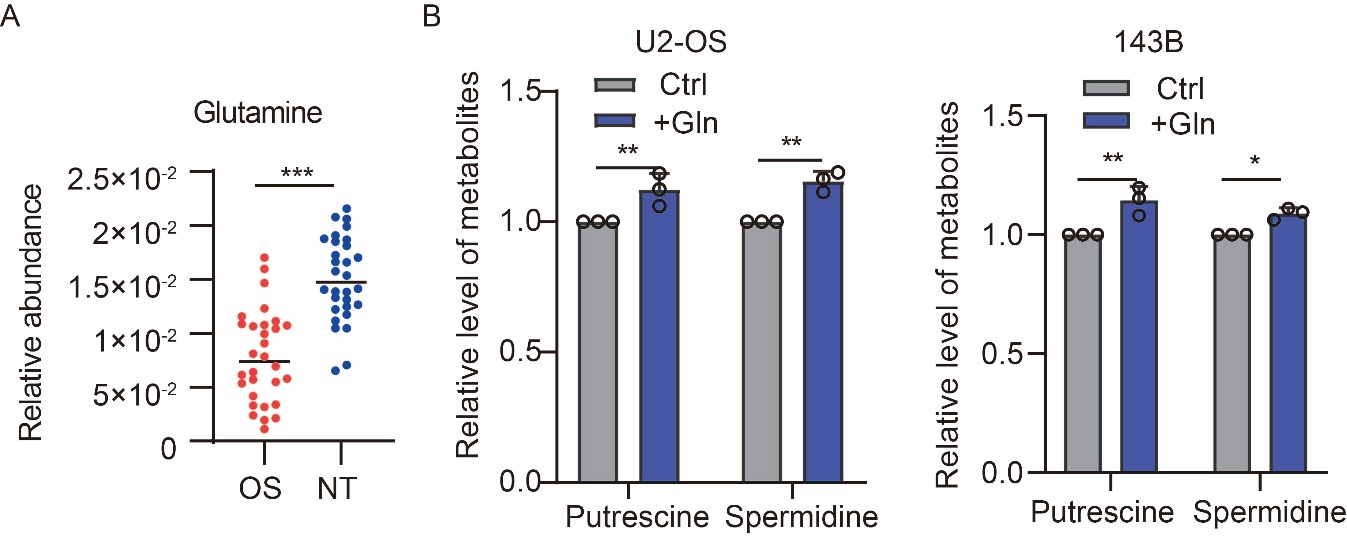


**Supplemental Figure 1. Role of Glutamine in Elevated Levels of Polyamines in Osteosarcoma (Related to Figure 1).**

A. Analysis of glutamine levels from non-targeted metabolomics in normal adjacent tissues (NT, n = 30) and osteosarcoma (OS, n = 30). Data are expressed as mean ± SD. Statistical analyses were conducted using a paired t-test.

B. Putrescine and spermidine levels in U2-OS and 143B cells following supplementation with 5 μM glutamine. Data are presented as mean ± SD for three replicates per group. Statistical significance determined by two-way ANOVA is denoted as follows: ***P ≤ 0.001, **P ≤ 0.01, *P ≤ 0.05, n.s.: not significant.

**Supplemental Figure 2**


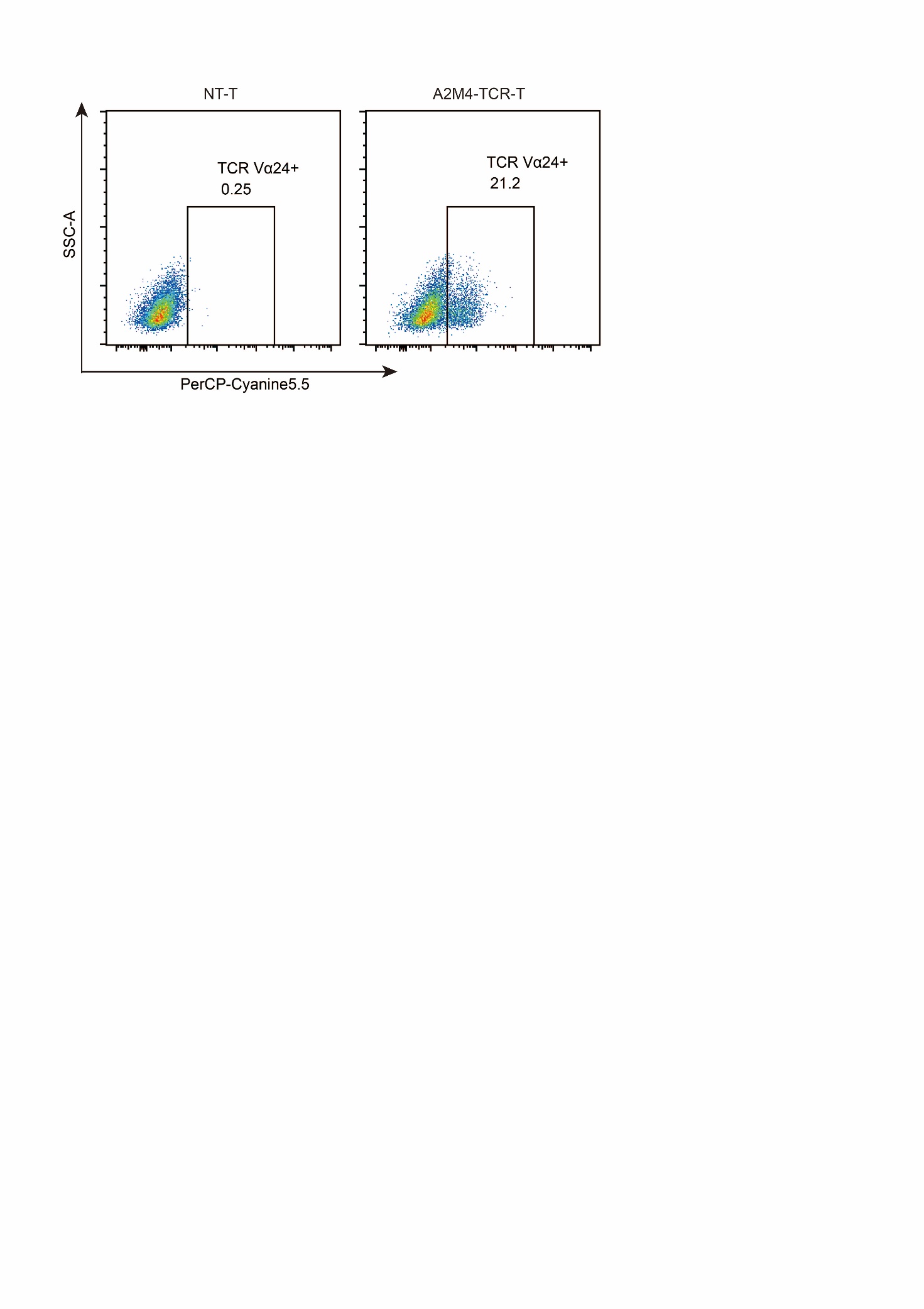


**Supplemental Figure 2. Evaluation of Transduction Efficiency of A2M4-TCR in Human T Cells (related to Figure 3).** Human T cells activated and then transduced with either an empty vector or A2M4-TCR were analyzed three days post-transduction using flow cytometry. Cells were stained with anti-Vα24-PerCP-Cyanine5.5 antibody to quantify A2M4-TCR expression.

**Supplemental Figure 3**


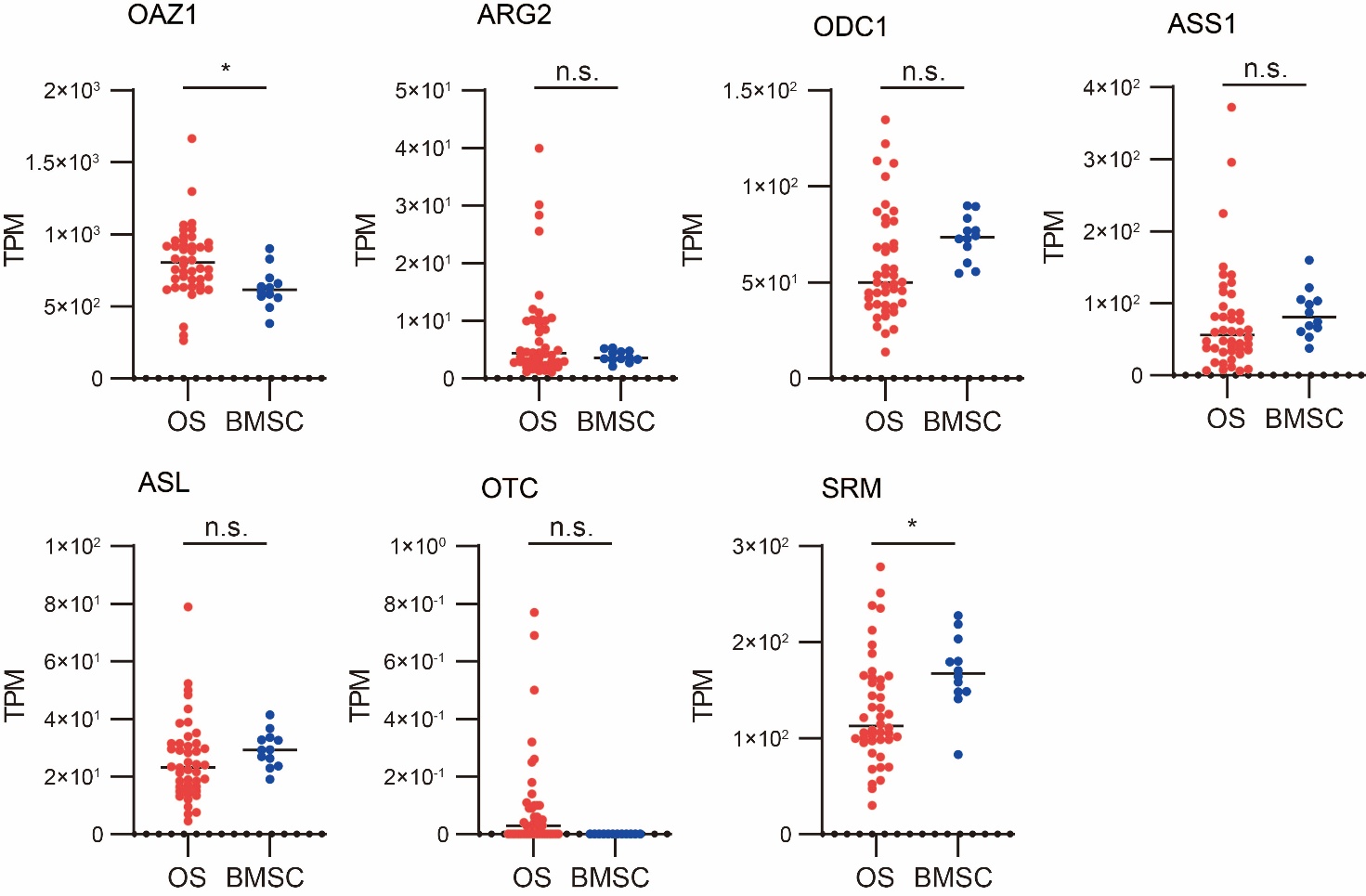


**Supplemental Figure 3. Differential Expression of Arginine Metabolism-Related Genes in Osteosarcoma and Bone Marrow Stromal Cells (related to Figure 4).** Expression levels were analyzed using data from publicly accessible datasets GSE87624 and E-MTAB-7925. Data are expressed as mean ± SD, with n = 12 for bone marrow stromal cells (BMSC) and n = 44 for osteosarcoma (OS). Statistical analyses were performed using an unpaired t-test. Levels of significance are indicated as ***P ≤ 0.001, **P ≤ 0.01, *P ≤ 0.05, n.s.: not significant.

**Supplemental Figure 4**


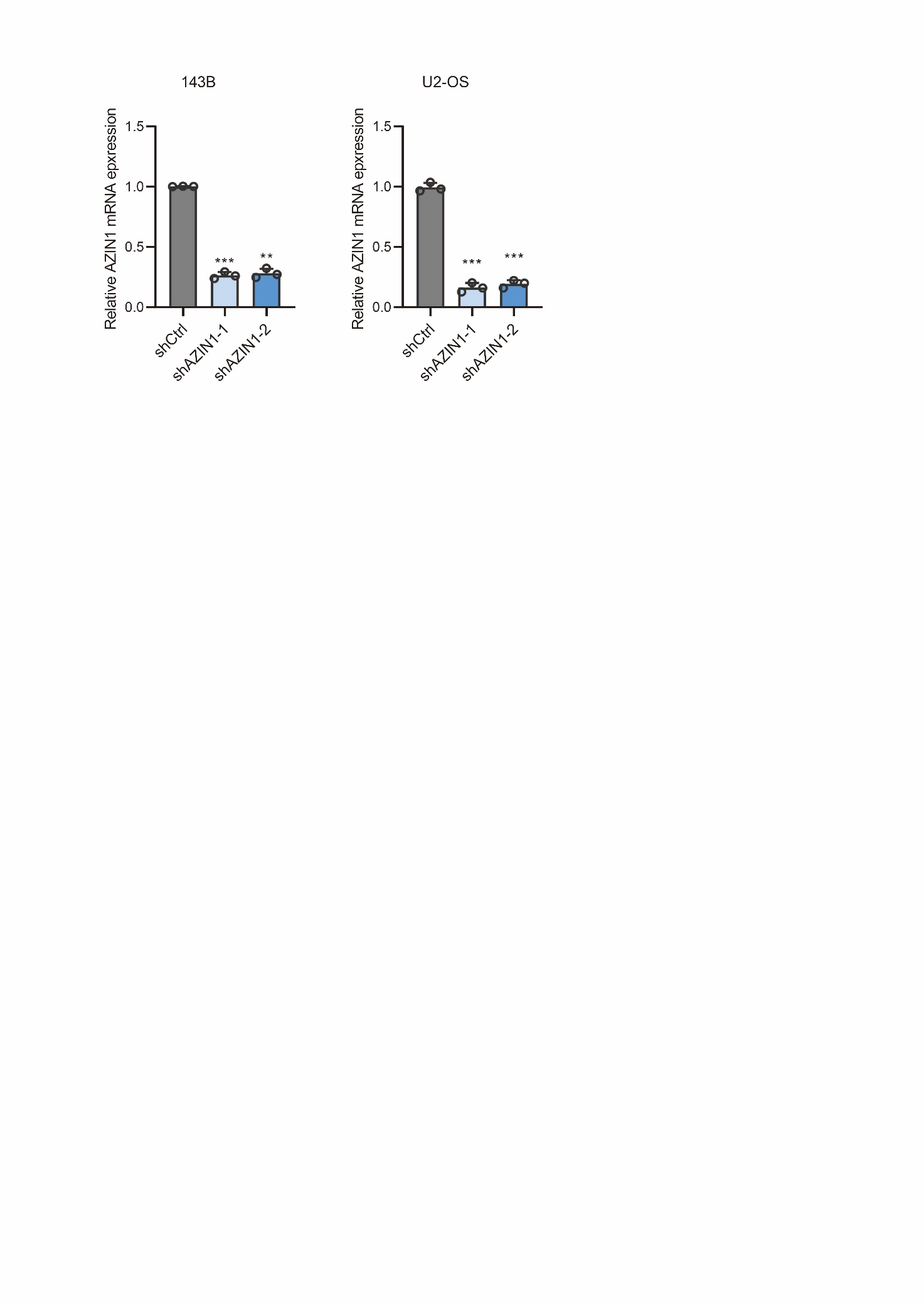


**Supplemental Figure 4. Validation of AZIN1 Knockdown in 143B and U2-OS Cell Lines (related to Figure 4).** Post-shRNA transduction, AZIN1 expression levels were measured via reverse transcription quantitative PCR (RT-qPCR). Data are expressed as mean ± SD, with three replicates per cell line (n = 3). Statistical analyses were performed using an unpaired t-test. Levels of significance are indicated as ***P ≤ 0.001, **P ≤ 0.01, *P ≤ 0.05, n.s.: not significant.

**Supplemental Figure 5**


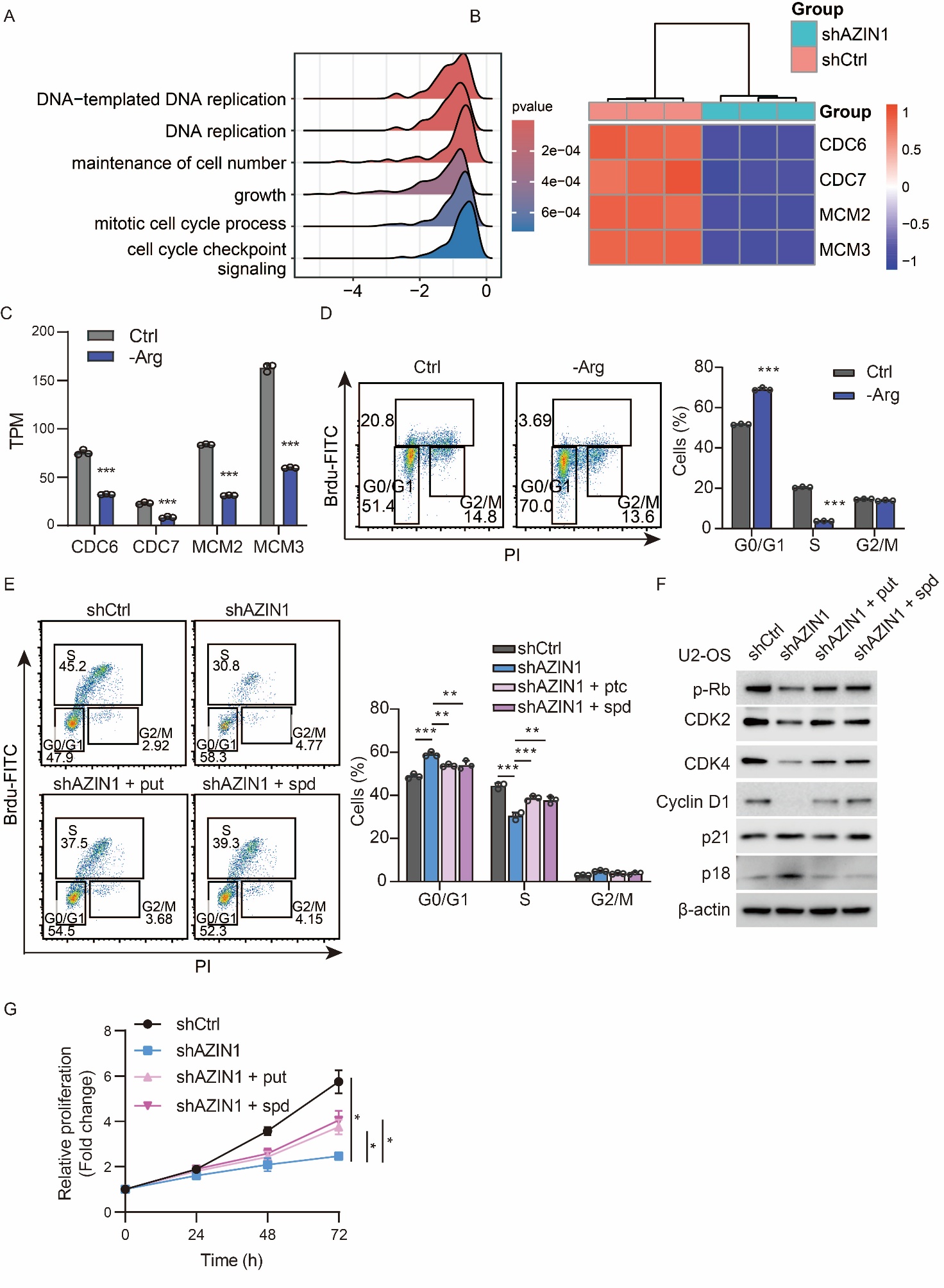


**Supplemental Figure 5. AZIN1 Facilitates Cell Cycle Progression in Osteosarcoma Cells in a Polyamine-Dependent Manner (Related to Figure 5).**

A. Gene Set Enrichment Analysis (GSEA) of RNA-seq data from U2-OS cells, illustrating the impact of *AZIN1* knockdown on cell cycle-related gene expression.

B. Heatmap illustrating gene expression levels of cell cycle-regulating genes in U2-OS cells with *AZIN1* knockdown.

C. Normalized expression levels of cell cycle-regulating genes from RNA-seq data of 143B cells in normal control medium versus arginine-deprived medium. Data are presented as mean ± SD (n = 3). Statistical analysis performed using an unpaired t-test.

D. Quantitative analysis of cell cycle changes in 143B cells cultured under the same conditions. Data are expressed as mean ± SD (n = 3). Statistical significance determined by one-way ANOVA.

E. Flow cytometry analysis of U2-OS cells with *AZIN1* knockdown supplemented with 10 μM putrescine (put) and 5 μM spermidine (spd), showing BrdU pulse-labeling and PI staining. Quantification of cell cycle phase distribution on the right. Data are expressed as mean ± SD (n = 3). Statistical analysis performed using one-way ANOVA.

F. Western blot analysis of cell cycle regulators in U2-OS cells after *AZIN1* knockdown and treatment with putrescine and spermidine.

G. Growth curves of U2-OS cells with *AZIN1* knockdown and subsequent supplementation with putrescine and spermidine. Data presented as mean ± SD (n = 3). Analyzed using one-way ANOVA.

Significance levels are indicated as follows: ***P ≤ 0.001, **P ≤ 0.01, *P ≤ 0.05, and n.s. for non-significant.

**Supplemental Figure 6**

**
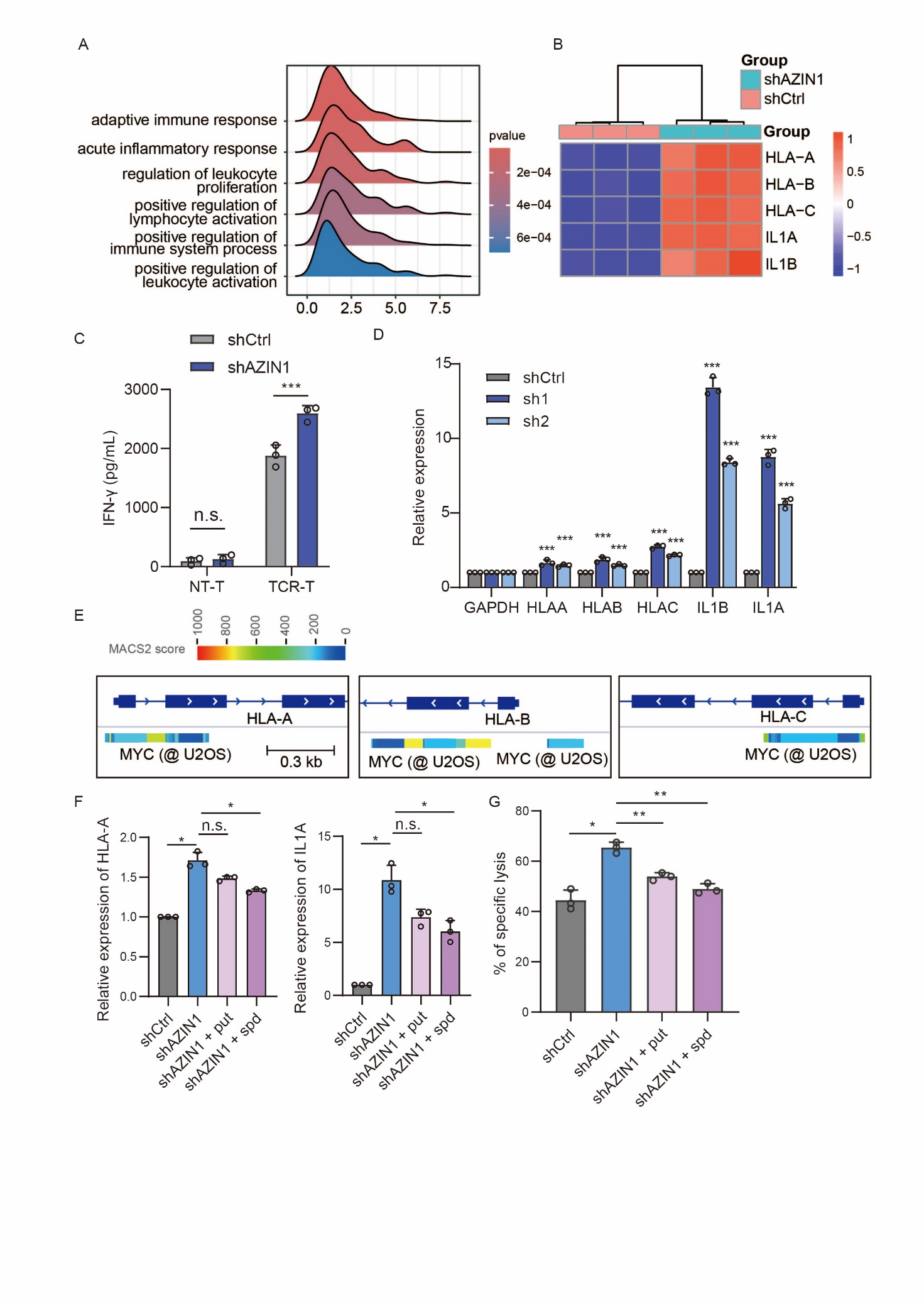
**

**Supplemental Figure 6. AZIN1-Mediated Polyamine Production Alters Osteosarcoma Immunogenicity and Enhances T Cell-Mediated Cytotoxicity (Related to Figure 6).**

A. Gene Set Enrichment Analysis (GSEA) from RNA-seq data of U2-OS cells with *AZIN1* knockdown, assessing changes in immune response-related gene expression.

B. Heatmap of transcriptional levels of genes regulating T cell responses in U2-OS cells with versus without *AZIN1* knockdown.

C. ELISA results for IFN-γ levels in T cells co-cultured with U2-OS cells under *AZIN1* knockdown and control conditions. Data presented as mean ± SD (n = 3). Analyzed using one-way ANOVA.

D. RT-qPCR analysis of T cell activation-related genes in U2-OS cells with or without *AZIN1* knockdown. Data expressed as mean ± SD (n = 3). Statistical analysis conducted using two-way ANOVA.

E. Representative IGV tracks showing MYC ChIP-seq binding on promoters of HLAs in U2-OS cells, derived from the ChIP-Atlas database.

F. RT-qPCR measurement of HLA-A and IL1A expression in U2-OS cells post-*AZIN1* knockdown and supplementation with 10 μM putrescine and 5 μM spermidine. Data are shown as mean ± SD (n = 3). Statistical evaluation using one-way ANOVA.

G. Assessment of TCR-T cell-mediated cytotoxicity against U2-OS cells with *AZIN1* knockdown and supplementation with putrescine or spermidine. Data are expressed as mean ± SD (n = 3). Statistical significance determined by one-way ANOVA.

Significance levels are indicated as follows: ***P ≤ 0.001, **P ≤ 0.01, *P ≤ 0.05, and n.s. for non-significant.

**Supplemental Figure 7**

**
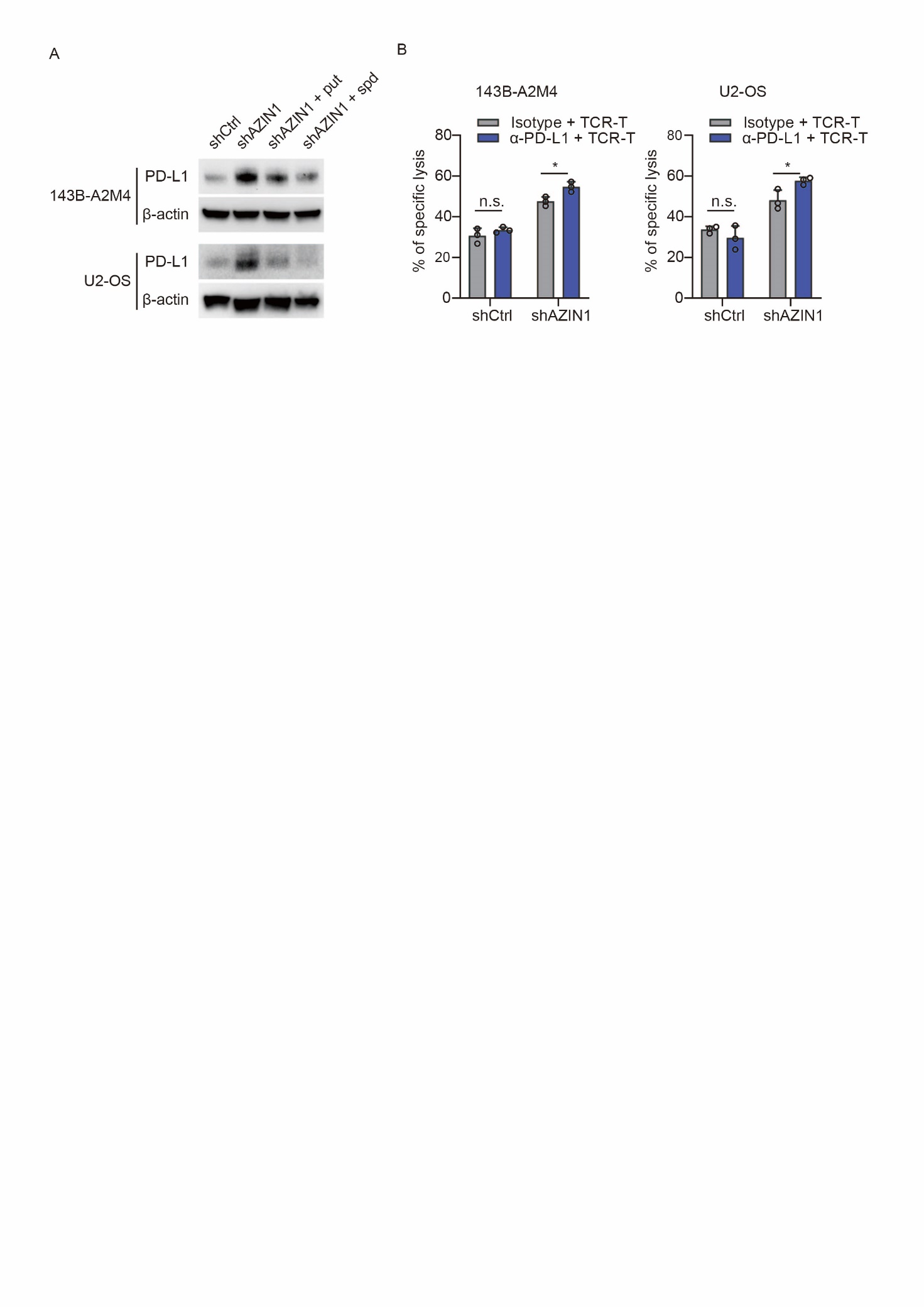
**

**Supplemental Figure 7. AZIN1-Mediated Polyamine Production Alters Immune Checkpoint Expression and Enhances the Efficacy of Immune Checkpoint Blockade (Related to Figure 6).**

A. Western blot analysis of PD-L1 levels in 143B-A2M4 and U2-OS osteosarcoma cells.

B. Evaluation of TCR-T cell-mediated cytotoxicity in 143B-A2M4 and U2-OS cells after *AZIN1* knockdown and treatment with 10 μg/mL Atezolizumab (α-PD-L1) or an isotype control antibody. Data are expressed as mean ± SD (n = 3). Statistical analysis conducted using two-way ANOVA.

Significance levels are indicated as follows: ***P ≤ 0.001, **P ≤ 0.01, *P ≤ 0.05, n.s.: not significant.

**Supplemental Table 1.** Sequences of shRNAs Used in This Study.

| Name | Sequence (5′-3′) |
| --- | --- |
| shAZIN1-1 | ATGAAGGAACAAACCTTGG |
| shAZIN1-2 | TGGAGAAATTGGCTTTACG |
| shCtrl | GCTTATCTGATGACCATGT |

**Supplemental Table 2.** List of Primers Used for PCR Amplifications.

| Name | Direction | Sequence (5′-3′) |
| --- | --- | --- |
| GADPH | Forward | GCACCGTCAAGGCTGAGAAC |
|  | Reverse | ATGGTGGTGAAGACGCCAGT |
| HLA-A | Forward | GCTCTCACTCCATGAGGTAT |
|  | Reverse | TCTGTGAGTGGGCCTTCAC |
| HLA-B | Forward | ACTGAGCTTGTGGAGACCAGA |
|  | Reverse | GCAGCCCCTCATGCTGT |
| HLA-C | Forward | CTGGCCCTGACCGAGACCTG |
|  | Reverse | CGCTTGTACTTCTGTGTCTCC |
| IL1A | Forward | AGATGCCTGAGATACCCAAAACC |
|  | Reverse | CCAAGCACACCCAGTAGTCT |
| IL1B | Forward | ATGATGGCTTATTACAGTGGCAA |
|  | Reverse | GTCGGAGATTCGTAGCTGGA |
| AZIN1 | Forward | GCCATTCTACACAGTGAAGTGC |
|  | Reverse | GAACAAGCAAATCCGGTTCCA |
